# Anatomical and Chemical Characterization of *Ulmus* Species from South Korea

**DOI:** 10.3390/plants10122617

**Published:** 2021-11-29

**Authors:** Woo-Sung Park, Hye-Jin Kim, Atif Ali Khan Khalil, Dong-Min Kang, Kazi-Marjahan Akter, Ji-Min Kwon, Yong-ung Kim, Xiang-Lan Piao, Kyung-Ah Koo, Mi-Jeong Ahn

**Affiliations:** 1 College of Pharmacy and Research Institute of Pharmaceutical Sciences, Gyeongsang National University, Jinju 52828, Korea; pws8822@gmail.com (W.-S.P.); dongminkang71@gmail.com (D.-M.K.); marjahan7silva@gmail.com (K.-M.A.); ellusian458@gmail.com (J.-M.K.); kk408842@gmail.com (K.-A.K.); 2 Food Science R&D Center, Kolmar BNH Co., Ltd., Seoul 06800, Korea; black200203@gmail.com; 3 Department of Biological Sciences, National University of Medical Sciences, Rawalpindi 46000, Pakistan; atif.khalil7799@gmail.com; 4 College of Herbal Bio-Industry, Daegu Haany University, Gyeongsan 38610, Korea; ykim@dhu.ac.kr; 5 School of Pharmacy, Minzu University of China, Beijing 100081, China; xlpiao@muc.edu.cn; 6 Azothbio Inc., Seongnam 13229, Korea

**Keywords:** *Ulmus* species, anatomical data, chemical differences, (-)-catechin-7-*O*-*β*-D-apiofuranoside, (-)-catechin-7-*O*-*α*-L-rhamnopyranoside

## Abstract

*Ulmus* species (*Ulmaceae*) are large deciduous trees distributed throughout Korea. Although their root and stem bark have been used to treat gastrointestinal diseases and wounds in folk medicine, commercial products are consumed without any standardization. Therefore, we examined anatomical and chemical differences among five *Ulmus* species in South Korea. Transverse sections of leaf, stem, and root barks were examined under a microscope to elucidate anatomical differences. Stem and root bark exhibited characteristic medullary ray and secretary canal size. Leaf surface, petiole, and midrib exhibited characteristic inner morphologies including stomatal size, parenchyma, and epidermal cell diameter, as well as ratio of vascular bundle thickness to diameter among the samples. Orthogonal projections to latent structures discriminant analysis of anatomical data efficiently differentiated the five species. To evaluate chemical differences among the five species, we quantified (-)-catechin, (-)-catechin-7-*O*-*β*-D-apiofuranoside, (-)-catechin-7-*O*-*α*-L-rhamnopyranoside, (-)-catechin-7-*O*-*β*-D-xylopyranoside, (-)-catechin-7-*O*-*β*-D-glucopyranoside, and (-)-catechin-5-*O*-*β*-D-apiofuranoside using high-performance liquid chromatography with a diode-array detector. (-)-Catechin-7-*O*-*β*-D-apiofuranoside content was the highest among all compounds in all species, and (-)-catechin-7-*O*-*α*-L-rhamnopyranoside content was characteristically the highest in *Ulmus parvifolia* among the five species. Overall, the *Ulmus* species tested was able to be clearly distinguished on the basis of anatomy and chemical composition, which may be used as scientific criteria for appropriate identification and standard establishment for commercialization of these species

## 1. Introduction

*Ulmus* genus (elm) of the *Ulmaceae* family is represented by deciduous arboreous trees with approximately 20 species distributed over the temperate zones of the Northern Hemisphere [1]. The dried root and stem bark have been used in China, Korea, and Japan as “Yugeunpi” and “Yubaekpi”, respectively, to treat gastrointestinal diseases and bedsores in traditional Korean medicine [2]. Meanwhile, the botanical origin of “Yugeunpi” and “Yubaekpi” used as folk medicine is unclear, and it is not known that the root or stem bark of which species has the most potent biological activity. Five *Ulmus* species, namely, *U. davidiana* var. *japonica* (Rehder) Nakai, *U. parvifolia* Jacq., *U. macrocarpa* Hance, *U. laciniata* (Trautv.) Mayr, and *U. pumila* L. are found in Korea [3]. The root bark extract of *U. davidiana* var. *japonica* possesses various pharmacological properties including neuroprotective, anti-inflammatory, anti-cancer, and antioxidant activities [4,5,6,7,8,9]. In addition, the stem and root bark extracts of *U. parvifolia* possess anti-allergic activity [10]. Moreover, the extract of and compounds isolated from *U. macrocarpa* root bark exhibit anti-inflammatory activity [11,12,13]. Several secondary metabolites such as catechin, catechin-7-*O*-α-L-rhamnopyranoside, catechin-3-*O*-α-L-rhamnopyranoside, lyoniside, (-)-lyoniresinol, (+)-lyoniresinol nudiposide, triterpenes, flavanones, and phenolics have been reported in the root bark of *U. davidiana* var. *japonica* [13,14,15,16]. Moreover, (-)-catechin-7-*O*-β-D-apiofuranoside, (-)-catechin, procyanidin B3, phloridzin, fraxetin, isovanillic acid, and vanillic acid have been isolated from the root bark of *U. parvifolia*, and sterol, sterol-glucoside, catechin-7-*O*-α-L-rhamnopyranoside, rutin, and isoquercetin have been isolated from its stem bark and leaves [17,18]. In addition, flavonoids and coumarins have been isolated from the root bark of *U. macrocarpa* [19,20].

While *U. parvifolia* belongs to Section *Microptelea*, the other four species belong to Section *Ulmus*. It is known that *Ulmus* species produce mucilage, and solitary pores or pore clusters with multiseriate medullary rays exist in the wood specimens of this genus [21,22]. The wood of *Ulmus* species has diffuse or semi-ring or ring porosity, one to eight seriate and homocellular rays, and prismatic crystals in axial parenchyma or enlarged cells [21]. The wood of the five *Ulmus* species in South Korea has ring porosity. *U. parvifolia* has 2–3 deep pores in earlywood zone and thick-walled fibers in latewood zone. *U. macrocarpa* has one deep pore in earywood zone. While more than three rows of earlywood vessels are found in *U. parvifolia* and *U. pumila*, one to three rows in the other three species. In an anatomical study on the bark of *Ulmus* species, it was reported that orderly arranged phloem fibers and parenchyma strands were found in the bark of *U. americana* seedlings, but significant intracellular space was not observed [23].

While previous anatomical studies such as comparative anatomy of *Ulmaceae*, wood anatomy of extant *Ulmaceae*, and that of 12 species and two varieties from *Ulmus* from China have been reported, along with their phytochemicals and biological activities, anatomical and chemical differences among the root and stem barks of the five *Ulmus* species in South Korea remain unexplored [21,22,23,24]. Therefore, the present study aimed to establish a standardization method to identify the botanical origin of biomedical materials on the basis of differences in the inner morphological and phytochemical characteristics of the five *Ulmus* species in Korea.

## 2. Results and Discussion

### 2.1. Anatomical Characteristics of the Leaf Surface

**Figure 1:**
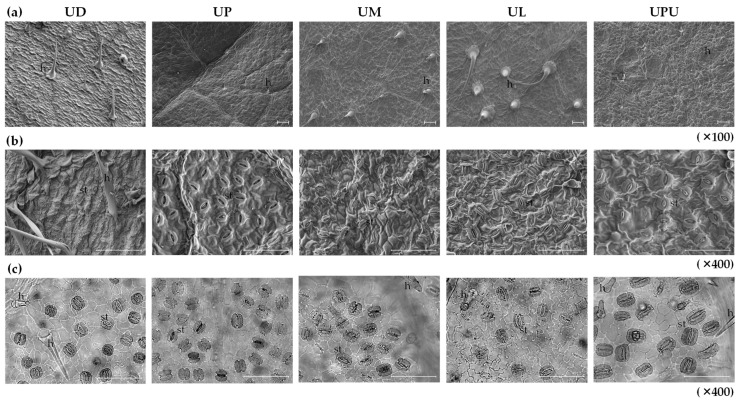
Photomicrographs of the leaf surfaces of the five *Ulmus* species tested. (**a**) Scanning electron microscopy (SEM) images of the adaxial leaf surface. (**b**) SEM images of the abaxial leaf surface. (**c**) Photomicrographs of the abaxial leaf surface. The white or black bars indicate 100 µm. *h*, hair; *st*, stomata. UD, *Ulmus davidiana* var. *japonica*; UP, *U. parvifolia*; UM, *U. macrocarpa*; UL, *U. laciniata*; UPU, *U. pumila*.

The adaxial and abaxial leaf surfaces of the five *Ulmus* species were subjected to scanning electron microscopy (SEM) (Figure 1a,b). Hairs were observed on both the leaf surfaces in all species, except on the abaxial leaf surface in *U. parvifolia*. They were unicellular pointed trichomes. Short uniseriate stalked trichomes with a multicellular head were also found in all species, except in *U. parvifolia*. This result is consistent with the previous report that multicellular trichomes were not founded in *U. americana*, *U. crassiflola*, *U. glabra*, *U. minor*, *U. laevis*, *U. parvifolia*, and *U. thomasii* [22]. There is no report on *U. davidiana* var. *japonica*, and *U. laciniata* showed longer hair and higher hair density on the adaxial leaf surface compared to the others. *U. parvifolia* showed short hair and the lowest hair density among the five species. Anomocytic stomata were observed only on the abaxial leaf surface of all species (Figure 1c). The number of stomata, but not the stomatal index, is significantly affected by factors such as growth, environment, maturity, veins, and hair [25,26]. The longest stomatal apparatus was found in *U. pumila* (30.0 ± 1.1 μm) and the shortest in *U. macrocarpa* (18.8 ± 1.4 μm). Likewise, the widest stomatal width was found in *U. pumila* (25.7 ± 1.2 μm), while the narrowest in *U. macrocarpa* (14.4 ± 1.5 μm). In this study, the stomatal index of *U. pumila* and *U. macrocarpa* was the highest (19.2 ± 0.2 and 18.8 ± 0.1, respectively) and the lowest was of *U. davidiana* var. *japonica* (11.4 ± 0.2) (Figure 1c). The freqeuncy of stomata was the highest in *U. davidiana* var. *japonica*, followed by *U. macrocarpa*, and the lowest in *U. parvifolia* (Table 1).

### 2.2. Anatomical Characteristics of the Leaf Midrib

**Figure 2:**
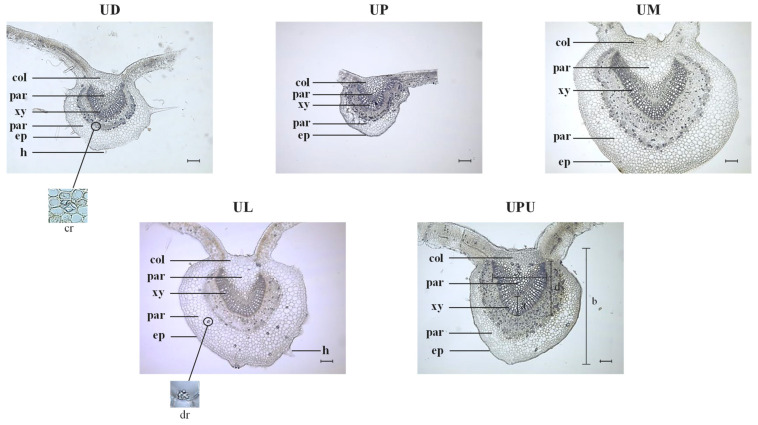
Photomicrographs of the leaf midrib of the five *Ulmus* species (×100). The black bars indicate 100 µm. *cr***,** crystal; *col*, collenchyma cells; *dr*, druse; *h*, hair; *ep*, epidermis; *par*, parenchyma cell; *xy*, xylem; a, vascular bundle thickness; b, midrib diameter; c, vascular bundle width; d, vascular bundle height. UD, *Ulmus davidiana* var. *japonica*; UP, *U. parvifolia*; UM, *U. macrocarpa*; UL, *U. laciniata*; UPU, *U. pumila*.

The vascular bundle of the midrib was U-shaped in all *Ulmus* species (Figure 2). The vascular bundle width was similar to vascular bundle height (Table 2). The widest epidermal cells in the adaxial part were found in *U. parvifolia* (20.1 ± 0.8 μm) and the narrowest were in *U. davidiana* var. *japonica* (11.5 ± 0.7 μm). The longest epidermal cells were observed in *U. parvifolia* (15.3 ± 1.5 μm) and the shortest in UPU and *U. davidiana* var. *japonica* (10.5 ± 1.4 and 9.9 ± 0.9 μm, respectively). The widest epidermal cells in the abaxial part were observed in *U. laciniata* (18.9 ± 1.4 μm) and the narrowest in *U. davidiana* var. *japonica* and *U. parvifolia* (14.0 ± 3.3 and 13.8 ± 1.3 μm, respectively). The longest epidermal cells were observed in *U. laciniata* and *U. macrocarpa* (14.9 ± 0.6 and 14.2 ± 3.5 μm, respectively). The longest diameter of collenchyma cells was observed in *U. laciniata* (38.6 ± 4.0 μm), and the shortest in *U. davidiana* var. *japonica* (15.0 ± 1.6 μm). Collenchyma cells support the stem near the epidermis and the parenchyma cells, helping the plant to grow straight [27]. The diameter of parenchyma cells in the cortex and pith was the longest in *U. laciniata* (29.8 ± 2.8 and 25.6 ± 2.2 μm, respectively). Higher ratio of vascular bundle thickness to midrib diameter was observed in *U. parvifolia* and *U. pumila* (0.17 ± 0.01 and 0.16 ± 0.01, respectively) than the others (Table 2). No significant differences in the ratio of vascular bundle width to vascular bundle height was found among the samples with the value of about one. Calcium oxalate or calcium carbonate crystals were usually present in plant tissues. Prismatic calcium oxalate crystals in the midrib of the *Ulmus* species exhibited a U-shaped distribution pattern along the collenchyma cells, and these crystals were the most abundant in *U. pumila* and the least abundant in *U. laciniata*. In *U. laciniata*, both prismatic crystals and druses were detected in parenchyma cells (Figure 2).

### 2.3. Anatomical Characteristics of the Petiole

**Figure 3:**
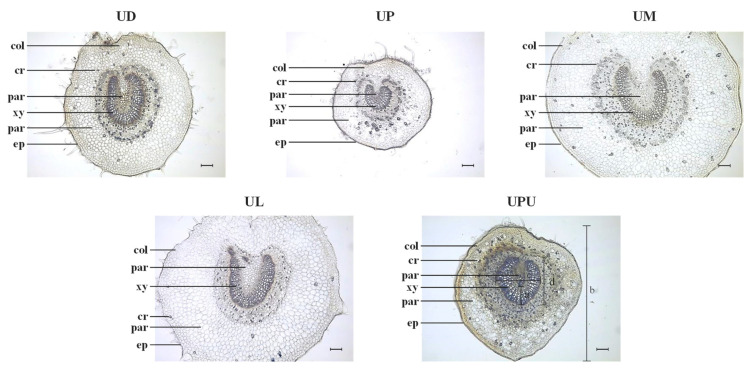
Photomicrographs of the petiole of the five *Ulmus* species (×100). The black bars mean 100 µm. *cr***,** crystal; *col*, collenchyma cells; *dr*, druse; *h*, hair; *ep*, epidermis; *par*, parenchyma cell; *xy*, xylem; a, vascular bundle thickness; b, midrib diameter; c, vascular bundle width; d, vascular bundle height. UD, *Ulmus davidiana* var. *japonica*; UP, *U. parvifolia*; UM, *U. macrocarpa*; UL, *U. laciniata*; UPU, *U. pumila*.

The petiole was round in all species. The widest epidermal cells in the adaxial part were observed in *U. macrocarpa* and *U. laciniata* (18.8 ± 3.2 and 18.3 ± 5.0 μm, respectively) and the narrowest in *U. pumila* (13.5 ± 1.0 μm). The longest epidermal cells in the adaxial part were observed in *U. macrocarpa* and *U. laciniata* (16.6 ± 3.5 and 13.9 ± 1.3 μm, respectively). The widest epidermal cells in the abaxial part were observed in *U. macrocarpa* and *U. laciniata* (24.5 ± 3.5 and 21.5 ± 3.2 μm, respectively), and the longest epidermal cells in this part were observed in *U. laciniata* and the shortest in *U. pumila*. Collenchyma cells were detected in all samples. A significant difference among the species was found in the diameter of collenchyma cells. While the values of *U. macrocarpa* and *U. laciniata* were more than 30 μm, they were less than 20 μm in *U. parvifolia* and *U. pumila*. The diameter of parenchyma cells in the cortex was the longest in *U. parvifolia* (23.5 ± 1.6 μm), and the diameter of parenchyma cells in the pith was the longest in *U. davidiana* var. *japonica* and *U. laciniata* (22.3 ± 5.5 and 20.3 ± 1.5 μm, respectively). The vascular bundle in the petiole was U-shaped in all species. The ratio of vascular bundle thickness to petiole diameter was the highest in *U. parvifolia* and *U. pumila* (0.10 ± 0.01 and 0.11 ± 0.02, respectively). Unlike leaf midrib, petiole showed significant difference in the ratio of vascular bundle width to vascular bundle height among the samples. While *U. parvifolia* showed square type of vascular bundle, oblong type of vascular bundle was observed in the others. *U. davidiana* var. *japonica* and *U. laciniata* showed the lowest ratio. Calcium oxalate crystals were detected, exhibiting a U-shape distribution pattern along collenchyma cells, in all species, except in *U. pumila*, in which prismatic crystals exhibited an O-shape distribution pattern. Interestingly, calcium oxalate crystals were mainly concentrated in the abaxial part of *U. parvifolia* but distributed throughout the parenchyma cells in *U. macrocarpa*. The abundance of druses was the highest in *U. pumila* and the lowest in *U. laciniata* (Figure 3 and Table 3).

### 2.4. Anatomical Characteristics of the Stem Bark

**Figure 4:**
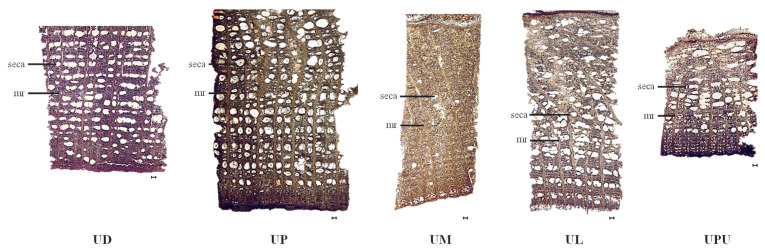
Photomicrographs of the stem bark of the five *Ulmus* species (×40). The black bars mean 100 µm. *mr*, medullary ray; *seca*, secretary canal. UD, *Ulmus davidiana* var. *japonica*; UP, *U. parvifolia*; UM, *U. macrocarpa*; UL, *U. laciniata*; UPU, *U. pumila*.

The medullary rays of the stem bark were straight in *U. davidiana* var. *japonica* and *U. parvifolia*, and were curved and closer to the epidermis in *U. macrocarpa*, *U. laciniata*, and *U. pumila*. The medullary ray was 2–4 cells thick in all species, with the highest frequency in *U. davidiana* var. *japonica* (4.3 ± 0.7 per 1 mm^2^). The length of the medullary ray cells was the longest in *U. laciniata* and *U. parvifolia* (51.4 ± 6.6 and 47.0 ± 7.2 μm, respectively) and the shortest in *U. macrocarpa* (20.0 ± 7.0 μm). The width of the medullary ray cells was the widest in *U. macrocarpa* (17.0 ± 1.8 μm), followed by *U. laciniata* (15.5 ± 0.5 μm). Secretory canals, which synthesize and store chemicals for defense against herbivores and pathogens [28], were uniformly distributed throughout the stem bark in all species, except in *U. macrocarpa*. It is known that *Ulmus* species produce mucilage, and solitary pores or pore clusters with multiseriate medullary rays exist in the wood specimens of this genus [21,22]. The frequency of secretary canals was the highest in *U. parvifolia* (23.4 ± 4.0%) and the lowest in *U. macrocarpa* (2.5 ± 4.0% in 1 mm^2^). The number of secretary canals was the highest in *U. parvifolia* and *U. davidiana* var. *japonica* (20.5 ± 2.7 and 18.4 ± 2.5, respectively). The widest the secretary canals were observed in *U. parvifolia* (167.9 ± 11.2 μm), while the longest secretary canals were observed in *U. parvifolia* and *U. pumila* (237.4 ± 28.8 and 224.5 ± 27.3 μm, respectively) (Figure 4 and Table 4).

### 2.5. Anatomical Characteristics of the Root Bark

**Figure 5:**
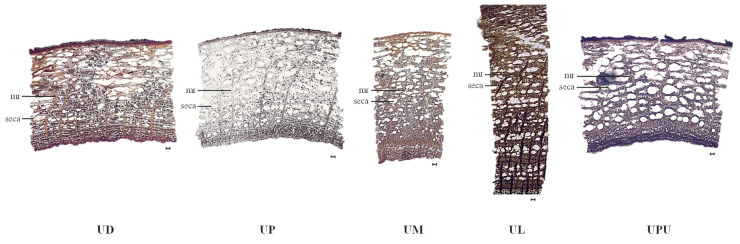
Photomicrographs of the root bark of the five *Ulmus* species (×40). The black bars indicate 100 µm. *mr*, medullary ray; *seca*, secretary canal. UD, *Ulmus davidiana* var. *japonica*; UP, *U. parvifolia*; UM, *U. macrocarpa*; UL, *U. laciniata*; UPU, *U. pumila*.

The medullary rays of the root bark, unlike those of the stem bark, were curved in all species, except in *U. laciniata*, in which they were disconnected (Figure 5). The medullary ray was 2–4 cells thick in all species, with the highest frequency in *U. laciniata* (3.6 ± 0.5 per 1 mm^2^). The longest medullary ray cells were observed in *U. pumila* (54.5 ± 5.7 μm), while the widest medullary ray cells were observed in *U. parvifolia* (19.0 ± 0.6 μm) and the narrowest in *U. laciniata*. Secretary canals were unevenly distributed and overlapped in the root bark, unlike those in the stem bark. The frequency of secretary canals was the highest in *U. pumila* (30.8 ± 0.2% per 1 mm^2^) and the lowest in *U. laciniata* and *U. davidiana* var. *japonica* (16.2 ± 1.4 and 16.8 ± 0.4% per 1 mm^2^, respectively). The number of secretary canals per square millimeter was the highest in *U. macrocarpa* (36.6 ± 8.1) and the lowest in *U. davidiana* var. *japonica* (13.9 ± 0.9). The longest secretary canals were observed in *U. pumila* (171.5 ± 1.7 μm) and the shortest in *U. davidiana* var. *japonica*. The widest secretary canals were observed in *U. pumila* (248.4 ± 15.5 μm) and the narrowest in *U. macrocarpa* and *U. laciniata* (Table 5).

### 2.6. Multivariate Statistical Analysis of Anatomical Data

**Figure 6:**
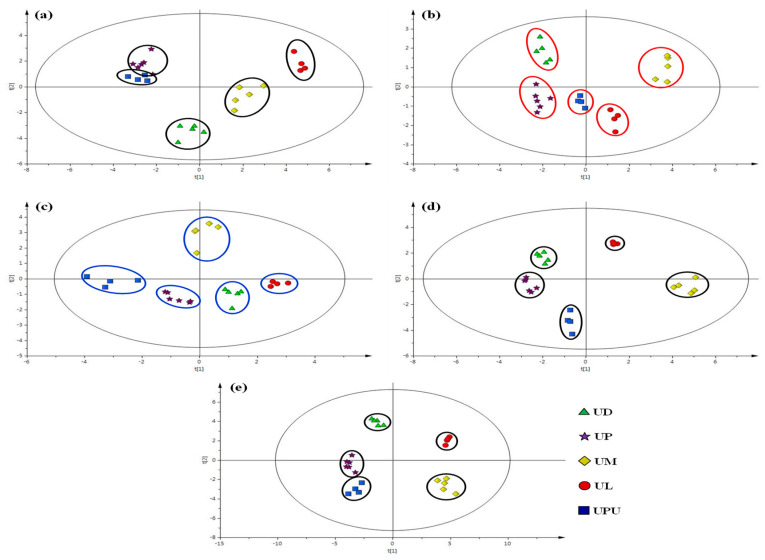
Orthogonal projections to latent structures–discriminant analysis of the anatomical data of five *Ulmus* species. Anatomical characteristics of leaf (**a**), stem bark (**b**), root bark (**c**), stem and root bark (**d**), and overall anatomical data (**e**). UD, *Ulmus davidiana* var. *japonica*; UP, *U. parvifolia*; UM, *U. macrocarpa*; UL, *U. laciniata*; UPU, *U. pumila*.

Orthogonal projections to latent structures–discriminant analysis (OPLS-DA) was performed to classify the five species on the basis of their anatomical data. Regarding leaf, the midrib and petiole presented characteristic sizes of the epidermal, parenchyma, and collenchyma cells on the abaxial and adaxial parts. Among the five *Ulmus* species, *U. davidiana* var. *japonica*, *U. macrocarpa*, and *U. laciniata* could be successfully distinguished, but *U. parvifolia* and *U. pumila* could not be distinguished and appeared to overlap with each other (Figure 6a). The leaves of *U. parvifolia* and *U. pumila* could not be distinguished from each other due to the similar ratio of vascular bundle thickness to diameter in the midrib and petiole. Regarding the stem and root bark, the five species presented similar values of frequency and size of the medullary ray cells but different values of the ratio, frequency, and size of the secretary canals. Therefore, *Ulmus* species could be distinguished using OPLS-DA of stem and root bark anatomical data (Figure 6b–d). Overall, the five *Ulmus* species could be clearly distinguished and classified on the basis of the anatomical features of the leaf, stem bark, and root bark (Figure 6e).

### 2.7. High-Performance Liquid Chromatography (HPLC) Profiles of the Five Ulmus Species

**Figure 7:**
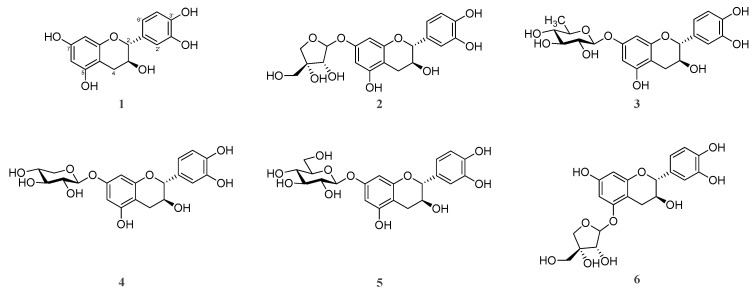
Chemical structures of compounds **1**–**6**.

**Figure 8:**
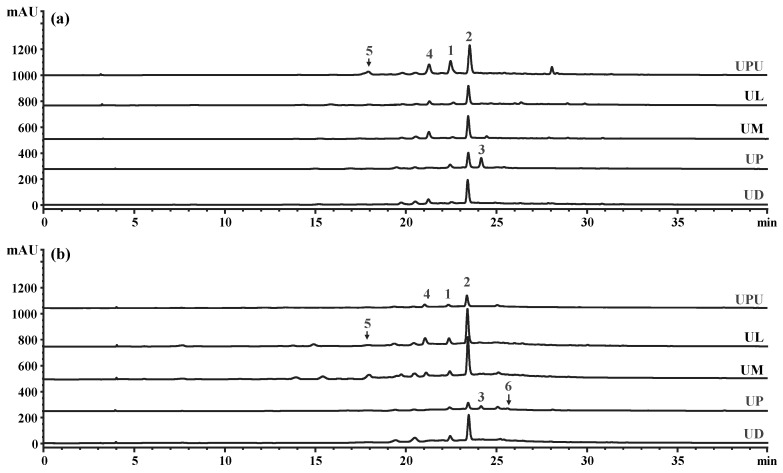
High-performance liquid chromatograms (280 nm) of the (**a**) stem bark and (**b**) root bark extracts of the five *Ulmus* species (10 mg/mL). **1**, (-)-catechin; **2**, (-)-catechin-7-*O*-*β*-D-apiofuranoside; **3**, (-)-catechin-7-*O*-*α*-L-rhamnopyranoisde; **4**, (-)-catechin-7-*O*-*β*-D-xylopyranoside; **5**, (-)-catechin-7-*O*-*β*-D-glucopyranoside; **6**, (-)-catechin-5-*O*-*β*-D-apiofuranoside.

Six compounds (**1**–**6**) corresponding to the major peaks in the HPLC chromatogram were isolated from 70% ethanol extracts of the bark of UP. The compounds were identified as (-)-catechin (**1**) and (-)-catechin glycosides of catechin-7-*O*-*β*-D-apiofuranoside (**2**), catechin-7-*O*-*α*-L-rhamnopyranoside (**3**), catechin-7-*O*-*β*-D-xylopyranoside (**4**), catechin-7-*O*-*β*-D-glucopyranoside (**5**), and catechin-5-*O*-*β*-D-apiofuranoside (**6**) on the basis of nuclear magnetic resonance spectrometry and mass spectrometry data (Figure 7 and Figures S1–S18) [29,30,31,32,33,34]. Compound **1** has various biological activities such as anti-microbial, anti-allergenic, anti-oxidant, anti-inflammatory and anti-cancer activities [14,35,36]. Compounds **2** and **3** showed hepatoprotective and anti-inflammatory activities, respectively [14,37]. Compounds **4** and **5** displayed anti-oxidant activity [36,38]. The isolated compounds were further used as external standards for quantification (Figure 8).

Considerable differences in the content of compounds **1**–**6** were observed among the five species. In the stem bark, the content of compound **1** was the highest in *U. pumila* (9.72 mg g^−1^∙dry weight (DW)^−1^), followed by *U. parvifolia* (2.78 mg g^−1^∙DW^−1^). The content of compound **2** was the highest among the six compounds in all species (23.62 to 21.21 mg g^−1^∙DW^−1^) in *U. pumila*, *U. davidiana* var. *japonica*, and *U. macrocarpa*; 15.95 mg g^−1^∙DW^−1^ in *U. laciniata*; and 14.08 mg g^−1^∙DW^−1^ in *U. parvifolia*. The content of compound **3** was the highest in *U. parvifolia* (6.81 mg g^−1^∙DW^−1^). The content of compound **4** was the highest in *U. pumila* (2.92 ± 0.34 mg g^−1^∙DW^−1^) and the lowest in *U. parvifolia* (0.49 ± 0.04 mg g^−1^∙DW^−1^). The content of compound **6** was the lowest among the six compounds in all species, and the content was the highest in *U. pumila* among the five species.

In the root bark, the content of compound **1** was the highest in *U. davidiana* var. *japonica* and *U. laciniata* (2.96 and 3.21 mg g^−1^∙DW^−1^, respectively), followed by *U. parvifolia* and *U. pumila* (1.15 and 1.26 mg g^−1^∙DW^−1^, respectively). The content of compound **2** was the highest among the six compounds in the root bark of all species, as observed in the stem bark. The content of compound **2** in the root bark was the highest in *U. macrocarpa* and *U. laciniata* (26.61 and 24.89 mg g^−1^∙DW^−1^, respectively). While the content of compound **2** was the lowest in *U. parvifolia*, the content of compound **3** was the highest in *U. parvifolia* (1.63 mg g^−1^∙DW^−1^), as observed in the stem bark. The content of compound **3** was similar among *U. davidiana* var. *japonica*, *U. laciniata*, and *U. macrocarpa* (0.51–0.57 mg g^−1^∙DW^−1^). The content of compound **4** was the highest in *U. laciniata*, followed by *U. macrocarpa*, and the content of compound **5** was the highest in *U. macrocarpa* (1.83 mg g^−1^∙DW^−1^), followed by *U. laciniata* (1.50 mg g^−1^∙DW^−1^). The content of compound **6** was the lowest in the root bark among the six compounds in all species, as observed in the stem bark. The content of compound **6** was the highest in *U. davidiana* var. *japonica* (0.46 mg g^−1^∙DW^−1^). In summary, the content of compound **2** was the highest among the six compounds in the stem and root bark of all species. Meanwhile, the content of compound **3** was the highest in the stem and root bark of *U. parvifolia* among the five species (Table 6). Although chemical taxonomy with popular flavonoids such myricetin and quercetin by classical paper chromatography has been accomplished on *Ulmus* species without *U. laciniata* and *U. davidiana* var. *japonica* [39], this is the first report on chemical differences by HPLC on the major secondary metabolites of five *Ulmus* species in South Korea.

## 3. Materials and Methods

### 3.1. Plant Materials and Reagents

Five *Ulmus* species were collected from various regions of Korea from April to October during 2017–2019. The samples were identified by Dr. Mi-Jeong Ahn, College of Pharmacy, Gyeongsang National University. The voucher specimens (PGSC-451–456, 461–464, 471–475, 481–484, and 491–494) were deposited in the herbarium of the College of Pharmacy, Gyeongsang National University (Table 7).

Glycerin (Junsei Chemicals Co., Ltd., Tokyo, Japan) was used to prepare specimens for anatomical examination. Ethanol (Daejung Chemicals and Metals Co., Ltd., Shiheung, Korea) was used for sample extraction. HPLC was performed using water and MeOH (Thermo Fisher Scientific Korea Ltd., Seoul, Korea). NMR solvents were purchased from Cambridge Isotope Laboratories, Inc. (Andover, MA, USA). Other reagents used were of high analytical grade.

### 3.2. Anatomical Study

Healthy and well-acclimatized samples (stem bark, root bark, midrib, and petiole of leaves) were obtained from the five *Ulmus* species and were preserved in 50% ethanol solution. Free hand or hand microtome sections with 20 to 40 μm-thickness were prepared using razor blades or a hand-held microtome (Euromex MT.5500, Arnhem, the Netherlands). Four to five sections were obtained from the middle part of the collected stem and root bark, lower part of midrib, and central part of petiole. The adaxial and abaxial leaf surfaces were analyzed using SEM (JSM-6380LV, Jeol, Tokyo, Japan) [38]. Eau de Javelle solution (Sigma, Minneapolis, MN, USA) was used to bleach the samples. Then, the samples were mounted in 100% glycerin or 50% glycerinated water on glass slides. All samples were observed under a light microscope (BX53F, Olympus, Tokyo, Japan), and photomicrographs were obtained using image processing software (IMT i-Solution Inc., Vancouver, BC, Canada) coupled to a video camera (PixeLINK, Ottawa, ON, Canada). Over five specimens of each species were analyzed to obtain representative characteristics, and five regions were measured on each photomicrograph.

Transverse sections of the stem and root bark were used to count the frequency of medullary rays and secretary canals in an area of 1 mm^2^. A range of 200 × 200 μm was selected to count the frequency of stomata and stomatal index on the abaxial leaf surface.

### 3.3. Sample Extraction and Compound Isolation

Dried stem bark of UP (300 g) was ground and extracted with 70% ethanol by sonication. The sample was concentrated in a rotary evaporator to obtain a crude extract (117 g). The crude extract was suspended in water and successively fractionated with *n*-hexane, dichloromethane, ethyl acetate, and *n*-butanol to yield *n*-hexane (2.0 g), CH_2_Cl_2_ (0.4 g), EtOAc (8.5 g), and *n*-BuOH (101.5 g) fractions. The EtOAc fraction was subjected to open silica column chromatography (CC) with a gradient elution system of CH_2_Cl_2_ and MeOH (100:0→0:100) to obtain 15 subfractions (fr.1–fr.15). Compound **1** (60 mg) was isolated from fr.6 using Sephadex LH-20 gel CC with methanol as the eluting solvent. Compound **2** (300 mg) was isolated from fr.9 through recrystallization. Subfraction fr.12 was further divided into four subfractions (fr.12.1–fr.12.4) using prep-HPLC. Subfraction fr.12.3 was subjected to Sephadex LH-20 gel CC with methanol as the eluting solvent to obtain compound **3** (200 mg). Subfraction fr.13 was further divided into eight subfractions (fr.13.1–fr.13.8) using prep-HPLC. Compounds **4** (5 mg), **5** (8 mg), and **6** (6 mg) were isolated from subfractions 13.2, 13.4, and 13.6, respectively, using Sephadex LH-20 gel CC with methanol as the eluting solvent. The prep-HPLC equipped with 312 pump, 155 detector, and GX-271 liquid handler of Gilson Company (Middleton, WI, USA) was used. The column was YMC Pack ODS-A (250 × 20 mm, 5 μm) equipped with a guard column. The flow rate was 4.0 mL/min, and eluent was detected at 280 nm. The gradient conditions using water and methanol were as follows: 15% methanol to 20% for the first 25 min, 20% to 90% for the next 10 min, 90% to 15% for 2 min, and then 15% kept for 5 min. For the compound identification, the nuclear magnetic resonance (NMR) spectroscopic data were obtained using Bruker DRX-300 and 500 spectrometers (Germany). EI, FAB, and ESI-MS were obtained using JMS-700 (Jeol) and Qtrap 4500 (Sciex, Framingham, MA, USA), respectively.

### 3.4. HPLC-DAD Profiling and Quantification

Dried stem and root barks were crushed in a grinder, and 250 mg of each sample was weighed. The powdered samples were extracted with 15 mL of 70% ethanol by sonicating it thrice for 60 min. The extract was centrifuged at 5752× *g* at 4 °C for 10 min (Eppendorf 5430R, Germany), and the supernatant was passed through a 0.45 μm PTFE syringe filter (Whatman, New York, NY, USA). Before HPLC analysis, the final volume was adjusted to 15 mL with 70% ethanol.

An Agilent 1260 series LC system equipped with an autosampler, a column oven, a binary pump, and a degasser (Agilent Technologies, Palo Alto, CA, USA) was used for the analysis. An aliquot (10 μL) of sample solution was directly injected on a Phenomenex Gemini C18 110A column (250 × 4.6 mm, 5 μm) equipped with a compatible guard column. Components were resolved by gradient elution using a 5% formic acid in water and methanol solvent system as follows, 10% to 80% methanol for the first 15 min, and then 80% to 10% for next 35 min. A conditioning phase was then used to return the column to the initial state for 5 min. The flow rate was 1.0 mL/min, and column temperature was 30 °C. The eluent was detected at 280 nm. LC chromatograms and in-line UV spectra were collected and analyzed using the Chemstation software (Agilent Technologies).

Quantification of compounds **1**–**6** was accomplished on the same LC condition used for profiling. Stock solutions of standard compounds were prepared with HPLC-grade methanol. For calibration curve, solutions were prepared by successive serial dilutions of the stock solution with methanol, and the final concentrations were 500, 250, 125, 62.5, 31.3, 15.6, 7.8, 3.9, 2.0, 1.0, and 0.5 μg/mL.

### 3.5. Statistical Analysis

All anatomical data of the stem bark, root bark, leaf, midrib, and petiole of each sample were subjected to OPLS-DA. OPLS-DA was performed using SIMCA (Ver. 13, Umetrics, Sweden). All values are expressed as mean ± standard deviation. One-way analysis of variance was performed using Excel (Microsoft, Redmond, WA, USA). Values with *p* < 0.05 were considered statistically significant.

## 4. Conclusions

Anatomical characteristics and phytochemical profiles significantly varied among the five *Ulmus* species from South Korea. Specifically, there were significant differences in the size of epidermal cells in the midrib and petiole of leaves, as well as the frequency of secretary canals, and moreover the number and size of medullary rays in stem and root bark. OPLS-DA of anatomical data could clearly distinguish the five *Ulmus* species tested. There were significant differences in the HPLC profiles of the six isolated compounds among the five samples. The content of compound **2** was the highest among the six compounds in stem and root bark and in all species. Meanwhile, the content of compound **3** was the highest in the stem and root bark of UP among the five species. Therefore, compound **2** can serve as an indicator for the standardization of *Ulmus* species, while compound **3** can serve as a valuable marker to distinguish UP from other *Ulmus* species. Anatomical characteristics and phytochemical profiles obtained in this study might be useful as reference to distinguish *Ulmus* plants in South Korea.

## Figures and Tables

**Table 1:** Anatomical characteristics of the abaxial leaf surface of the five *Ulmus* species.

Parameters	*U. davidiana* var. *japonica*	*U. parvifolia*	*U. macrocarpa*	*U. laciniata*	*U. pumila*
Stomatal length (μm)	21.3 ± 1.7 ^c^	26.0 ± 1.2 ^b^	18.8 ± 1.4 ^d^	26.8 ± 2.6 ^ab^	30.0 ± 1.1 ^a^
Stomatal width (μm)	16.9 ± 0.9 ^d^	19.3 ± 1.1 ^c^	14.4 ± 1.5 ^e^	21.2 ± 1.4 ^b^	25.7 ± 1.2 ^a^
Stomatal index (%)	11.4 ± 0.2 ^d^	13.5 ± 0.1 ^b^	18.8 ± 0.1 ^a^	12.5 ± 0.1 ^c^	19.2 ± 0.2 ^a^
Frequency of stomata	34.4 ± 5.1 ^a^	18.1 ± 0.7 ^b^	29.0 ± 2.2 ^a^	16.8 ± 0.9 ^c^	11.0 ± 0.8 ^d^

Data are expressed as mean ± SD (*n* > 3) of five independent experiments. Different upper letters in the same line indicate a significant difference (*p* < 0.05) among samples. A range of 200 μm × 200 μm was selected to count stomatal index and frequency of stomata.

**Table 2:** Anatomical characteristics of the leaf midrib of the five *Ulmus* species.

Parameters	*U. davidiana* var. *japonica*	*U. parvifolia*	*U. macrocarpa*	*U. laciniata*	*U. pumila*
Width of epidermal cells in adaxial part (μm)	11.5 ± 0.7 ^d^	20.1 ± 0.8 ^a^	16.0 ± 2.2 ^cb^	17.3 ± 1.2 ^b^	13.6 ± 0.7 ^c^
Length of epidermal cells in adaxial part (μm)	9.9 ± 0.9 ^b^	15.3 ± 1.5 ^a^	14.9 ± 2.1 ^a^	15.6 ± 1.6 ^a^	10.5 ± 1.4 ^b^
Width of epidermal cells in abaxial part (μm)	13.8 ±1.3 ^b^	15.6 ± 1.4 ^ab^	17.4 ± 5.6 ^ab^	18.9 ± 1.4 ^a^	14.0 ± 3.3 ^b^
Length of epidermal cells in abaxial part (μm)	12.1 ± 1.1 ^b^	12.1 ± 0.5 ^b^	14.2 ± 3.5 ^ab^	14.9 ± 0.6 ^a^	11.4 ± 2.1 ^b^
Diameter of collenchyma cells (μm)	15.0 ± 1.6 ^d^	21.5 ± 1.6 ^c^	32.0 ± 7.2 ^ab^	38.6 ± 4.0 ^a^	26.0 ± 3.6 ^b^
Diameter of parenchyma cells in cortex (μm)	20.2 ± 2.6 ^b^	19.4 ± 1.6 ^b^	22.8 ± 2.0 ^ab^	25.6 ± 2.2 ^a^	24.1 ± 0.8 ^a^
Diameter of parenchyma cells in pith (μm)	23.7 ± 1.6 ^b^	22.2 ± 2.7 ^bc^	28.2 ± 3.9 ^a^	29.8 ± 2.8 ^a^	20.1 ± 0.9 ^c^
Ratio of vascular bundle thickness to midrib diameter *	0.10 ± 0.01 ^b^	0.17 ± 0.01 ^a^	0.10 ± 0.01 ^b^	0.10 ± 0.01 ^b^	0.16 ± 0.01 ^a^
Ratio of vascular bundle width to vascular bundle height *	1.02 ± 0.18 ^a^	1.05 ± 0.02 ^a^	1.12 ± 0.27 ^a^	1.14 ± 0.18 ^a^	0.94 ± 0.07 ^a^

Data are expressed as mean ± SD (*n* > 3) of five independent experiments. Different upper letters in the same line indicate a significant difference (*p* < 0.05) among samples. * Ratio of vascular bundle thickness to midrib diameter, and ratio of vascular bundle width to vascular bundle height were calculated from a/b and c/d, respectively, in Figure 2.

**Table 3:** Anatomical characteristics of the petiole of the five *Ulmus* species.

Parameters	*U. davidiana* var. *japonica*	*U. parvifolia*	*U. macrocarpa*	*U. laciniata*	*U. pumila*
Width of epidermal cells in adaxial part (μm)	16.4 ± 1.2 ^ab^	15.5 ± 0.7 ^b^	18.8 ± 3.2 ^a^	18.3 ± 5.0 ^a^	13.5 ± 1.0 ^c^
Length of epidermal cells in adaxial part (μm)	14.4 ± 1.6 ^a^	9.3 ± 0.5 ^b^	13.9 ± 1.3 ^a^	16.6 ± 4.1 ^a^	9.4 ± 1.1 ^b^
Width of epidermal cells in abaxial part (μm)	15.2 ± 2.1 ^b^	16.1 ± 0.4 ^b^	21.5 ± 3.2 ^a^	24.7 ± 3.5 ^a^	14.0 ± 2.2 ^b^
Length of epidermal cells in abaxial part (μm)	13.5 ± 2.3 ^b^	12.5 ± 0.4 ^b^	15.1 ± 2.5 ^ab^	19.4 ± 3.1 ^a^	10.2 ± 1.2 ^c^
Diameter of collenchyma cells (μm)	22.2 ± 0.7 ^c^	17.7 ± 5.5 ^c^	31.5 ± 2.2 ^b^	37.3 ± 2.3 ^a^	17.9 ± 2.3 ^c^
Diameter of parenchyma cells in cortex (μm)	20.3 ± 1.5 ^a^	14.2 ± 3.3 ^b^	17.5 ± 0.6 ^ab^	22.3 ± 5.5 ^a^	16.8 ± 1.9 ^ab^
Diameter of parenchyma cells in pith (μm)	29.1 ± 1.9 ^c^	23.5 ± 1.1 ^d^	38.6 ± 2.4 ^b^	42.9 ± 1.7 ^a^	26.7 ± 3.7 ^cd^
Ratio of vascular bundle thickness to petiole diameter *	0.08 ± 0.01 ^b^	0.10 ± 0.01 ^a^	0.07 ± 0.01 ^b^	0.07 ± 0.01 ^b^	0.11 ± 0.02 ^a^
Ratio of vascular bundle width to vascular bundle height *	0.71 ± 0.03 ^c^	1.03 ± 0.02 ^a^	0.94 ± 0.05 ^b^	0.80 ± 0.09 ^c^	0.94 ± 0.01 ^b^

Data are expressed as mean ± SD (*n* > 3) of five independent experiments. Different upper letters in the same line indicate a significant difference (*p* < 0.05) among samples. * Ratio of vascular bundle thickness to petiole diameter, and ratio of vascular bundle width to vascular bundle height were calculated from a/b and c/d, respectively, in Figure 3.

**Table 4:** Anatomical characteristics of the stem bark of the five *Ulmus* species.

Parameters	*U. davidiana* var. *japonica*	*U. parvifolia*	*U. macrocarpa*	*U. laciniata*	*U. pumila*
Number of layers in medullary rays	2.2 ± 0.2 ^b^	3.2 ± 0.4 ^a^	4.1 ± 1.2 ^a^	3.2 ± 0.4 ^a^	3.5 ± 0.1 ^a^
Frequency of medullary rays (in 1 mm^2^)	4.3 ± 0.7 ^a^	3.3 ± 0.5 ^b^	2.9 ± 0.4 ^b^	2.9 ± 0.5 ^b^	2.8 ± 0.3 ^b^
Length of medullary ray cells (µm)	35.9 ± 1.6 ^b^	47.0 ± 7.2 ^a^	20.2 ± 7.0 ^c^	51.4 ± 6.6 ^a^	37.4 ± 3.0 ^b^
Width of medullary ray cells (µm)	12.6 ± 0.9 ^b^	13.3 ± 1.7 ^b^	17.0 ± 1.8 ^a^	15.3 ± 0.5 ^ab^	11.7 ± 2.0 ^b^
Ratio of secretary canals (%, in 1 mm^2^)	20.7 ± 2.8 ^ab^	23.4 ± 4.0 ^a^	2.5 ± 0.5 ^c^	17.6 ± 3.0 ^b^	20.0 ± 0.9 ^ab^
Frequency of secretary canals (in 1 mm^2^)	18.4 ± 2.5 ^a^	20.5 ± 2.7 ^a^	2.7 ± 0.6 ^c^	3.2 ± 0.4 ^c^	15.5 ± 0.6 ^b^
Length of secretary canals (µm)	145.9 ± 9.6 ^b^	167.9 ± 11.2 ^a^	89.6 ± 11.0 ^e^	108.6 ± 11.8 ^d^	133.6 ± 1.9 ^c^
Width of secretary canals (µm)	169.4 ± 41.3 ^b^	237.4 ± 28.8 ^a^	129.8 ± 5.5 ^c^	191.9 ± 6.5 ^ab^	224.5 ± 27.3 ^a^

Data are expressed as mean ± SD (*n* > 3) of five independent experiments. Different upper letters in the same line indicate a significant difference (*p* < 0.05) among samples.

**Table 5:** Anatomical characteristics of the root bark of the five *Ulmus* species.

Parameters	*U. davidiana* var. *japonica*	*U. parvifolia*	*U. macrocarpa*	*U. laciniata*	*U. pumila*
Number of layers in medullary rays	2.0 ± 0.2 ^b^	2.6 ± 0.4 ^ab^	2.2 ± 0.3 ^b^	3.2 ± 0.7 ^a^	2.2 ± 0.1 ^b^
Frequency of medullary rays (in 1 mm^2^)	2.9 ± 0.3 ^b^	2.5 ± 0.5 ^bc^	1.7 ± 0.04 ^c^	3.6 ± 0.5 ^a^	2.1 ± 0.2 ^c^
Length of medullary ray cells (µm)	40.6 ± 2.5 ^b^	41.2 ± 2.5 ^b^	41.8 ± 5.0 ^b^	41.5 ± 5.4 ^b^	54.5 ± 5.7 ^a^
Width of medullary ray cells (µm)	16.2 ± 1.3 ^bc^	19.0 ± 0.6 ^a^	18.3 ± 2.9 ^ab^	15.0 ± 0.5 ^c^	16.1 ± 0.7 ^b^
Ratio of secretary canal (%, in 1 mm^2^)	16.8 ± 0.4 ^c^	21.4 ± 1.4 ^b^	24.2 ± 5.6 ^ab^	16.2 ± 1.4 ^c^	30.8 ± 0.2 ^a^
Frequency of secretary canals (in 1 mm^2^)	13.9 ± 0.9 ^c^	18.9 ± 1.1 ^b^	36.6 ± 8.1 ^a^	19.6 ± 2.4 ^b^	21.4 ± 2.6 ^b^
Length of secretary canals (µm)	116.6 ± 5.6 ^c^	157.3 ± 5.4 ^b^	100.8 ± 11.5 ^d^	103.7 ± 3.1 ^d^	171.5 ± 1.7 ^a^
Width of secretary canals (µm)	192.0 ± 2.3 ^b^	243.2 ± 10.0 ^a^	163.3 ± 7.7 ^c^	163.5 ± 2.7 ^c^	248.4 ± 15.5 ^a^

Data are expressed as mean ± SD (*n* > 3) of five independent experiments. Different upper letters in the same line indicate a significant difference (*p* < 0.05) among samples.

**Table 6:** Contents of six compounds in the stem and root bark of the five *Ulmus* species.

Parts	Species	1	2	3	4	5	6
Stem bark	UD	1.31 ± 0.06 ^c^	22.72 ± 2.39 ^a^	1.07 ± 0.19 ^b^	1.52 ± 0.19 ^b^	0.67 ± 0.00 ^d^	0.32 ± 0.05 ^b^
	UP	2.78 ± 0.21 ^b^	14.08 ± 1.03 ^b^	6.81 ± 0.51 ^a^	0.49 ± 0.04 ^d^	1.06 ± 0.07 ^b^	0.25 ± 0.01 ^c^
	UM	1.40 ± 0.09 ^c^	21.21 ± 3.31 ^a^	1.19 ± 0.05 ^b^	1.83 ± 0.20 ^b^	0.75± 0.12 ^d^	0.31 ± 0.01 ^b^
	UL	1.44 ± 0.03 ^c^	15.95 ± 0.33 ^b^	0.65 ± 0.02 ^c^	0.81 ± 0.06 ^c^	0.93 ± 0.07 ^c^	0.21 ± 0.01 ^d^
	UPU	9.72 ± 1.08 ^a^	23.62 ± 0.71 ^a^	0.62 ± 0.00 ^c^	2.92 ± 0.34 ^a^	5.65 ± 0.57 ^a^	0.45 ± 0.04 ^a^
Root bark	UD	2.96 ± 0.11 ^a^	20.02 ± 0.48 ^b^	0.56 ± 0.02 ^c^	0.24 ± 0.01 ^e^	0.66 ± 0.02 ^e^	0.46 ± 0.01 ^a^
	UP	1.15 ± 0.07 ^c^	4.40 ± 1.38 ^d^	1.63 ± 0.17 ^a^	0.13 ± 0.02 ^d^	0.74 ± 0.10 ^d^	0.18 ± 0.03 ^d^
	UM	2.63 ± 0.03 ^b^	26.61 ± 0.95 ^a^	0.51 ± 0.05 ^c^	0.93 ± 0.07 ^b^	1.83 ± 0.14 ^a^	0.32 ± 0.01 ^b^
	UL	3.21 ± 0.10 ^a^	24.89 ± 0.26 ^a^	0.57 ± 0.04 ^c^	1.78 ± 0.10 ^a^	1.50 ± 0.15 ^b^	0.25 ± 0.02 ^c^
	UPU	1.26 ± 0.01 ^c^	7.94 ± 0.26 ^c^	1.13 ± 0.01 ^b^	0.51 ± 0.02 ^c^	0.95 ± 0.15 ^c^	0.32 ± 0.05 ^b^

Data are expressed as mean ± SD (mg/g dry weight) of three independent experiments. Different upper letters in the same column indicate a significant difference (*p* < 0.05) among the samples. UD, *Ulmus davidiana* var. *japonica*; UP, *U. parvifolia*; UM, *U. macrocarpa*; UL, *U. laciniata*; UPU, *U. pumila*. **1**, (-)-catechin; **2**, (-)-catechin-7-*O*-*β*-D-apiofuranoside; **3**, (-)-catechin-7-*O*-*α*-L-rhamnopyranoisde; **4**, (-)-catechin-7-*O*-*β*-D-xylopyranoside; **5**, (-)-catechin-7-*O*-*β*-D-glucopyranoside; **6**, (-)-catechin-5-*O*-*β*-D-apiofuranoside.

**Table 7:** A list of *Ulmus* plants collected from Korea.

Botanical Name	Collection Place (Latitude, Longitude)	Specimen No.
*Ulmusdavidiana* var. *japonica* (Rehder) Nakai	Jinju (35°09′03.2″ N, 128°17′40.3″ E) Sancheong (35°19′18.7″ N,127°45′19.9″ E) Hadong (35°14′17.5″ N, 127°42′19.0″ E) Pocheon (37°45′23.7″ N, 127°10′03.8″ E)	PGSC-451–456
*Ulmus parvifolia* Jacq.	Jinju (35°12′54.9″ N, 128°04′07.3″ E) (35°13′00.3″ N, 128°04′09.0″ E) (35°09′27.4″ N, 128°17′43.9″ E) Busan (35°22′02.4″ N, 129°13′64.1″ E) (35°22′02.6″ N, 129°13′20.7″ E)	PGSC-461–464
*Ulmus macrocarpa* Hance	Bonghwa (36°47′15.3″ N, 128°54′24.0″ E) Yeongwol (37°12′31.1″ N, 128°25′58.1″ E) Pocheon (37°45′23.3″ N, 127°09′58.7″ E)	PGSC-471–475
*Ulmus laciniata* (Trautv.) Mayr	Hadong (35°14′59.2″ N, 127°42′29.1″ E) (35°15′00.9″ N, 127°42′30.2″ E) Pocheon (37°45′19.0″ N, 127°09′51.9″ E)	PGSC-481–484
*Ulmus pumila* L.	Jeongseon (37°18′53.3″ N, 128°37′30.0″ E) (37°21′47.4″ N, 128°36′34.6″ E) Pocheon (37°45′18.0″ N, 127°09′57.5″ E)	PGSC-491–494

## Data Availability

Date is contained within the article and Supplementary Material.

## Supplementary Materials

The following are available online at https://www.mdpi.com/article/10.3390/plants10122617/s1, Figure S1, The EI-MS spectrum of compound **1**. Figure S2, The ^1^H-NMR spectrum of compound **1**. Figure S3, The ^13^C-NMR spectrum of compound **1**. Figure S4, The FAB-MS spectrum of compound **2**. Figure S5, The ^1^H-NMR spectrum of compound **2**. Figure S6, The ^13^C-NMR spectrum of compound **2**. Figure S7, The FAB-MS spectrum of compound **3**. Figure S8, The ^1^H-NMR spectrum of compound **3**. Figure S9, The ^13^C-NMR spectrum of compound **3**. Figure S10, The ESI-MS spectrum of compound **4**. Figure S11, The ^1^H-NMR spectrum of compound **4**. Figure S12, The ^13^C-NMR spectrum of compound **4**. Figure S13, The ESI-MS spectrum of compound **5**. Figure S14, The ^1^H-NMR spectrum of compound **5**. Figure S15, The ^13^C-NMR spectrum of compound **5**. Figure S16, The ESI-MS spectrum of compound **6**. Figure S17, The ^1^H-NMR spectrum of compound **6**. Figure S18, The ^13^C-NMR spectrum of compound **6**. ^1^H and ^13^C NMR assign data of isolated compounds.

## References

[1] Abbasi S., Hosseini S.M., Khorasani N., Karbassi A. (2018). Responses of the morphological traits of elm (*Ulmus* minor ‘umbraculifera’) leaves to air pollution in urban areas (A case study of Tehran Metropolitan city, Iran). Appl. Ecol. Environ. Res..

[2] Jun C.D., Pae H.O., Kim Y.C., Jeong S.J., Yoo J.C., Lee E.J., Choi B.M., Chae S.W., Park R.K., Chung H.T. (1998). Inhibition of nitric oxide synthesis by butanol fraction of the methanol extract of *Ulmus davidiana* in murine macrophages. J. Ethnopharmacol..

[3] Lee Y.N. (2006). Flora of Korea.

[4] Lee Y., Park H., Ryu H.S., Chun M., Kang S., Kim H.-S. (2007). Effects of elm bark (*Ulmus davidiana* var. *japonica*) extracts on the modulation of immunocompetence in mice. J. Med. Food.

[5] Zheng M.S., Li G., Li Y., Seo C.-S., Lee Y.-K., Jung J.-S., Song N.-K., Bae H.-B., Kwak S.-H., Chang H.-W. (2011). Protective constituents against sepsis in mice from the root barks of *Ulmus davidiana* var. *japonica*. Arch. Pharm. Res..

[6] Baek I., Im L.-H., Park C., Choi Y.H. (2015). Anti-cancer potentials of *Rhus verniciflua* stokes, *Ulmus davidiana* var. *japonica* Nakai and *Arsenium sublimatum* in human gastric cancer AGS cells. J. Life Sci..

[7] Kim H.-S., Cho J.-H., Lee J.-M., Lee C.-H., Jang J.-B., Lee K.-S. (2010). Experimental studies on antimetastatic and immunomodulating effects of *Ulmus davidiana*. J. Orient. Obstet. Gynecol..

[8] Ahn J.J., Lee J.S., Yang K.M. (2014). Ultrafine particles of *Ulmus davidiana* var. *japonica* induce apoptosis of gastric cancer cells via activation of caspase and endoplasmic reticulum stress. Arch. Pharm. Res..

[9] Zheng M.S., Lee Y.-K., Li Y., Hwangbo K., Lee C.-S., Kim J.-R., Lee S.K.-S., Chang H.-W., Son J.-K. (2010). Inhibition of DNA topoisomerases I and II and cytotoxicity of compounds from *Ulmus davidiana* var. *japonica*. Arch. Pharm. Res..

[10] Kim S.P., Lee S.J., Nam S.H., Friedman M. (2016). Elm tree (*Ulmus parvifolia*) bark bioprocessed with mycelia of shiitake (*Lentinus edodes*) mushrooms in liquid culture: Composition and mechanism of protection against allergic asthma in mice. J. Agric. Food Chem..

[11] Kwon J.-H., Kim S.-B., Park K.-H., Lee M.-W. (2011). Antioxidative and anti-inflammatory effects of phenolic compounds from the roots of *Ulmus macrocarpa*. Arch. Pharm. Res..

[12] Kang M.C., Yumnam S., Park W.S., So H.M., Kim K.H., Shin M.C., Ahn M.-J., Kim S.Y. (2019). *Ulmus parvifolia* accelerates skin wound healing by regulating the expression of MMPs and TGF-β. J. Clin. Med..

[13] Kim H.-J., Yeom S.-H., Kim M.-K., Shim J.-G. (2004). Nitric oxide and prostaglandin E2 synthesis inhibitory activities of flavonoids from the barks of *Ulmus macrocarpa*. Nat. Prod. Sci..

[14] Zheng M.S., Yang J.-H., Li Y., Li X., Chang H.-W., Son J.-K. (2010). Anti-inflammatory activity of constituents isolated from *Ulmus davidiana* var. *japonica*. Biomol. Ther..

[15] Lee M.K., Sung S.H., Lee H.S., Cho J.H., Kim Y.C. (2001). Lignan and neolignan glycosides from *Ulmus davidiana* var. *japonica*. Arch. Pharm. Res..

[16] Lee M.K., Kim Y.C. (2001). Five novel neuroprotective triterpene esters of *Ulmus davidiana* var. *japonica*. J. Nat. Prod..

[17] Moon Y.H., Rim G.R. (1995). Studies on the constituents of *Ulmus parvifolia*. Kor. J. Pharmacogn..

[18] Kim S.H., Hwang K.T., Park J.C. (1992). Isolation of flavonoids and determination of rutin from the leaves of *Ulmus parvifolia*. Kor. J. Pharmacogn..

[19] So H.M., Yu J.S., Khan Z., Subedi L., Ko Y.-J., Lee I.K., Park W.S., Chung S.J., Ahn M.-J., Kim S.Y. (2019). Chemical constituents of the root bark of *Ulmus davidiana* var. *japonica* and their potential biological activities. Bioorg. Chem..

[20] Kwoun Y.M., Lee J.H., Lee M.W. (2002). Phenolic compounds from bark of *Ulmus macrocarpa* and its antioxidative activities. Kor. J. Pharmacogn..

[21] Wheeler E., Manchester S. (2007). Review of the wood anatomy of extant Ulmaceae as context for new reports of late Eocene *Ulmus* woods. Bull. Geosci..

[22] Sweitzer E.M. (1971). The comparative anatomy of Ulmaceae. J. Arnold Arbor..

[23] Yamamoto F., Angeles G., Kozlowski T.T. (1987). Effect of ethrel on stem anatomy of *Ulmus americana* seedlings. IAWA J..

[24] Li H., Zhang J., Gao Z., Li Y. (2007). Wood anatomy of 12 species and 2 varieties from *Ulmus* of China. J. Henan For. Sci. Technol..

[25] Kokate C.K. (1994). Practical Pharmacognosy.

[26] Salisbury E.J. (1927). On the causes and ecological significance of stomatal frequency, with special reference to the woodland flora. Philos. Trans. R. Soc. London B.

[27] Leroux O. (2012). Collenchyma, a versatile mechanical tissue with dynamic cell walls. Ann. Bot..

[28] Fahn A., Every R.F. (1974). Ultrastructure of the secretory ducts of *Rhus glabra* L.. Amer. J. Bot..

[29] Abd El-Razek M.H. (2007). NMR assignments of four catechin epimers. Asian J. Chem..

[30] Na M.K., An R.B., Lee S.M., Min B.S., Kim Y.H., Bae K.H., Kang S.S. (2002). Antioxidant compounds from the stem bark of *Sorbus commixta*. Nat. Prod. Sci..

[31] Lee A.H., Lee M.W. (1995). Tannins from *Rubus coreanum*. Kor. J. Pharmacogn..

[32] Inoshiri S., Sasaki M., Kohda H., Otsuka H., Yamasaki K. (1987). Aromatic glycosides from *Berchemia racemosa*. Phytochemistry.

[33] Foo L.Y., Karchesy J.J. (1989). Polyphenolic glycosides from *Douglas fir* inner bark. Phytochemistry.

[34] Son B.W., Park J.H., Zee O.-P. (1989). Catechin glycoside from *Ulmus davidiana*. Arch. Pharm. Res..

[35] Bae J., Kim N., Shin Y., Kim S.-Y., Kim Y.-J. (2020). Activity of catechins and their applications. Biomed. Dermatol..

[36] Jung M.J., Heo S.-I., Wang M.-H. (2010). HPLC analysis and antioxidant activity of *Ulmus davidiana* and some flavonoids. Food Chem..

[37] Park Y.J., Kim D.M., Jeong M.H., Yu J.S., So H.M., Bang I.J., Kim H.R., Kwon S.-H., Kim K.H., Chung K.H. (2019). (–)-Catechin-7-*O*-*β*-D-apiofuranoside inhibits hepatic stellate cell activation by suppressing the STAT3 signaling pathway. Cells.

[38] Cui E.-J., Song N.-Y., Shrestha S., Chung I.-S., Kim J.-Y., Jeong T.-S., Baek N.-I. (2012). Flavonoid glycosides from cowpea seeds (*Vigna sinensis* K.) inhibit LDL oxidation. Food Sci. Biotechnol..

[39] Bate-Smith E.C., Richens R.H. (1973). Flavonoid chemistry and taxonomy in *Ulmus*. Biochem. Syst..

