# Supplementary material for: Anatomical and Chemical Characterization of Ulmus Species from South Korea
Source: Plants (Basel). 2021 Nov 29;10(12):2617. doi: 10.3390/plants10122617 (PMC8707370; doi:10.3390/plants10122617)
Supplement: Supplementary file 1 [file plants-10-02617-s001.zip › plants-1422405-supplementary.pdf]

Supplementary Data

Anatomical and Chemical Characterization of *Ulmus* Species from South Korea

Woo Sung Park, Hye-Jin Kim, Atif Ali Khan Khalil, Kazi-Marjahan Akter, Dong-Min Kang, Ji-Min Kwon, Yong-ung Kim, Xiang-Lan Piao, Kyung Ah Koo and Mi-Jeong Ahn

Figure S1. The EI-MS spectrum of compound **1** (70 eV)

Figure S2. The ^{1}H-NMR spectrum of compound **1** (300 MHz, CD_3OD)

Figure S3. The ^{13}C-NMR spectrum of compound **1** (125 MHz, CD_3OD)

Figure S4. The FAB-MS spectrum of compound **2** (*m*-NBA)

Figure S5. The ^{1}H-NMR spectrum of compound **2** (300 MHz, CD_3OD)

Figure S6. The ^{13}C-NMR spectrum of compound **2** (125 MHz, CD_3OD)

Figure S7. The FAB-MS spectrum of compound **3** (*m*-NBA)

Figure S8. The ^{1}H-NMR spectrum of compound **3** (500 MHz, CD_3OD)

Figure S9. The ^{13}C-NMR spectrum of compound **3** (125 MHz, CD_3OD)

Figure S10. The ESI-MS spectrum of compound **4**

Figure S11. The ^{1}H-NMR spectrum of compound **4** (500 MHz, CD_3OD)

Figure S12. The ^{13}C-NMR spectrum of compound **4** (125 MHz, CD_3OD)

Figure S13. The ESI-MS spectrum of compound **5**

Figure S14. The ^{1}H-NMR spectrum of compound **5** (500 MHz, CD_3OD)

Figure S15. The ^{13}C-NMR spectrum of compound **5** (125 MHz, CD_3OD)

Figure S16. The ESI-MS spectrum of compound **6**

Figure S17. The ^{1}H-NMR spectrum of compound **6** (500 MHz, CD_3OD)

Figure S18. The ^{13}C-NMR spectrum of compound **6** (125 MHz, CD_3OD)

^{1}H and ^{13}C NMR assign data of isolated compounds

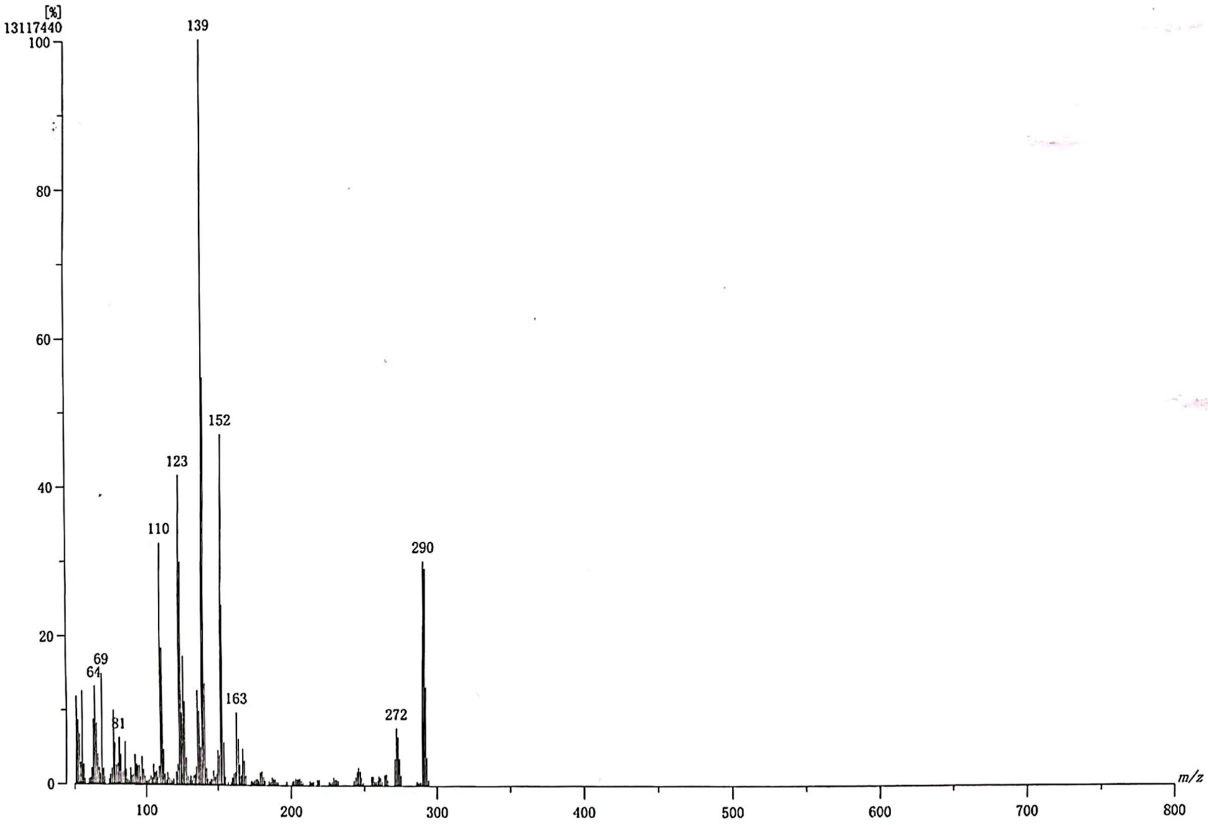

Figure S1. The EI-MS spectrum of compound **1** (70 eV)

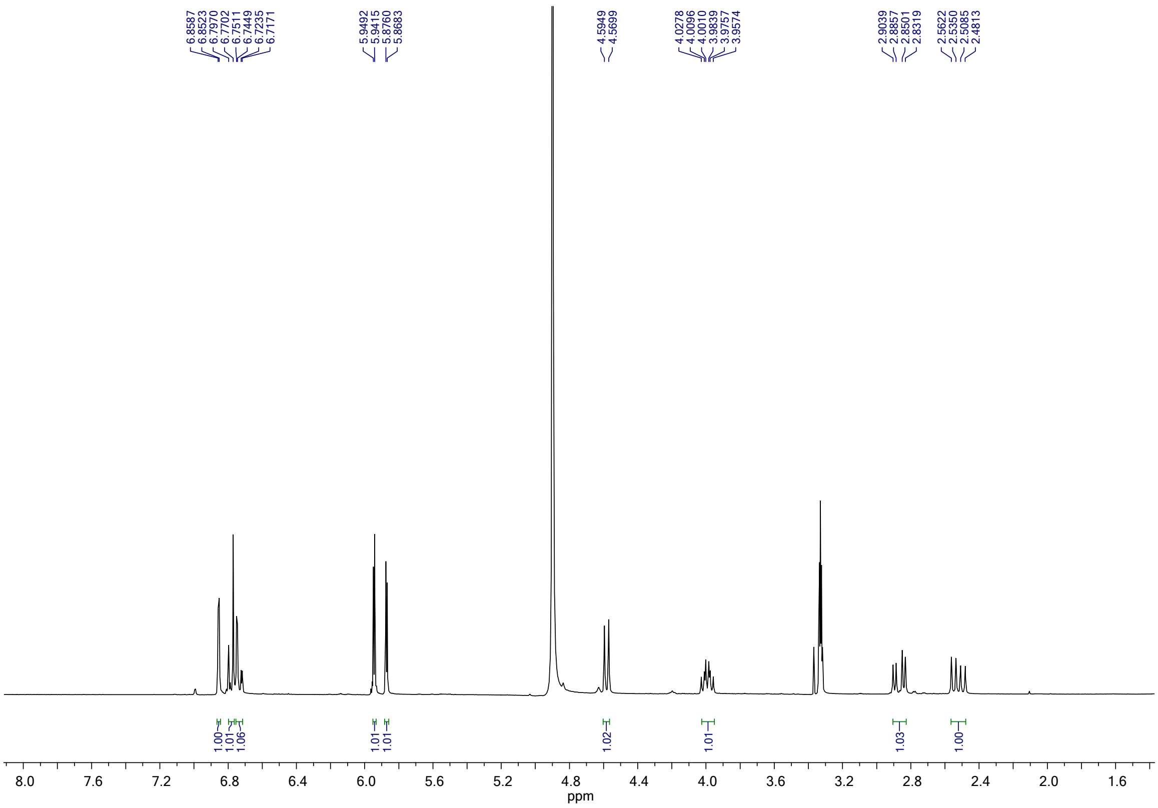

Figure S2. The ^{1}H-NMR spectrum of compound **1** (300 MHz, CD_3OD)

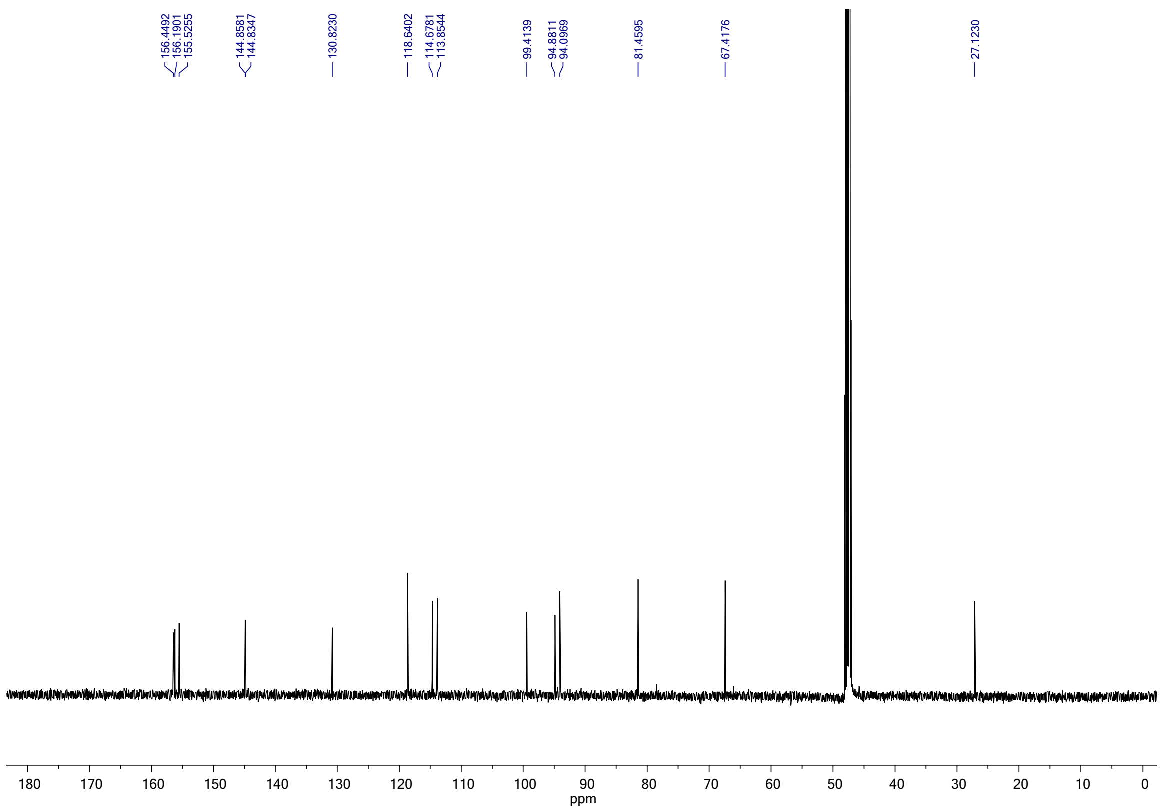

Figure S3. The ^{13}C-NMR spectrum of compound **1** (125 MHz, CD_3OD)

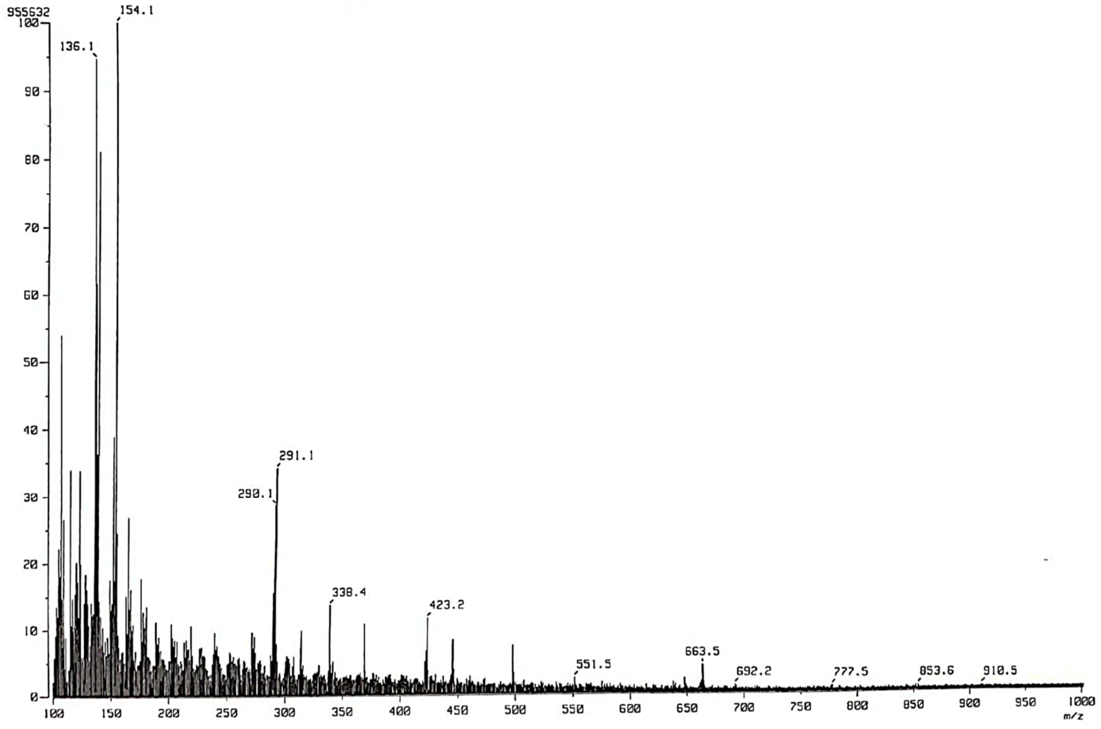

Figure S4. The FAB-MS spectrum of compound **2** (*m*-NBA)

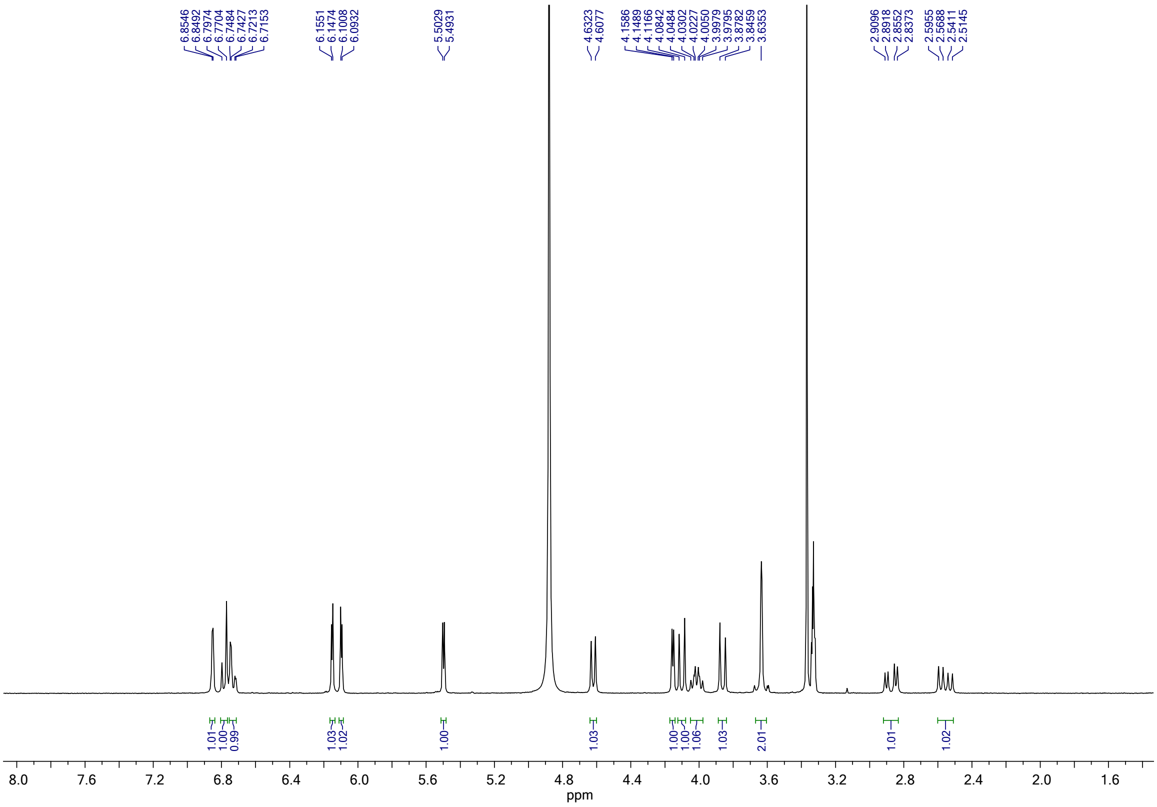

Figure S5. The ^{1}H-NMR spectrum of compound **2** (300 MHz, CD_3OD)

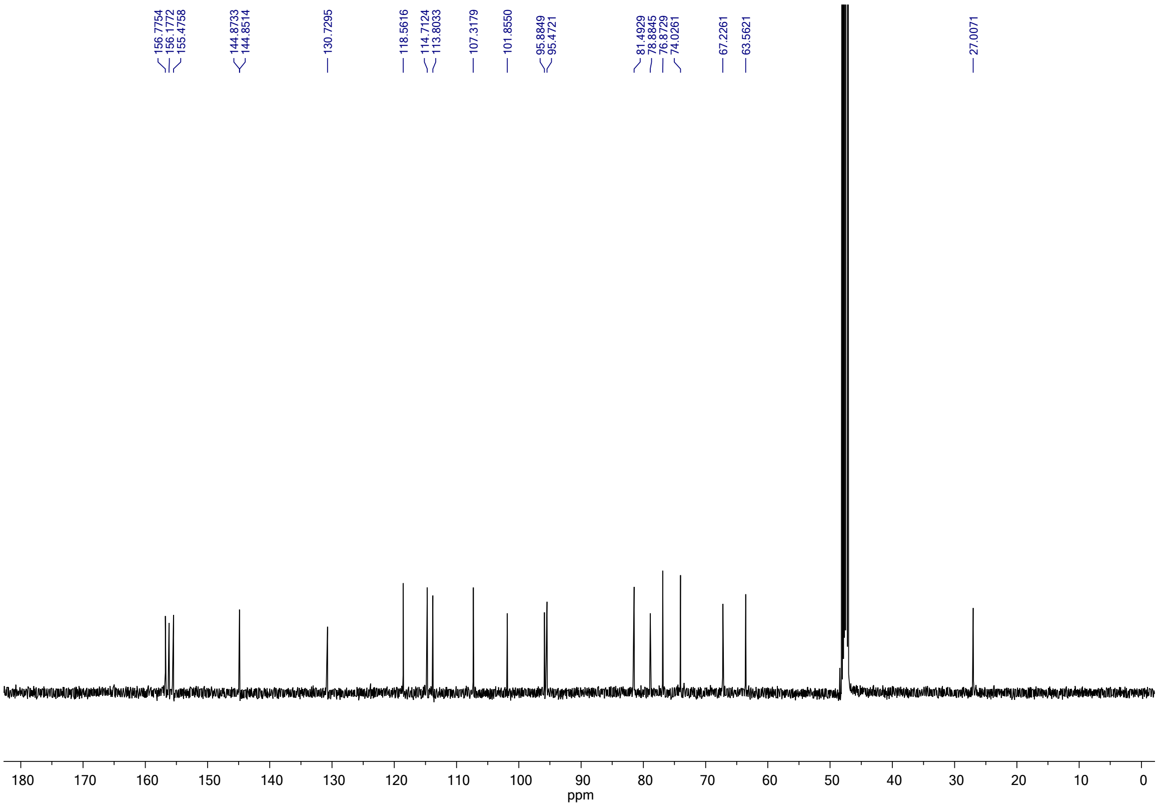

Figure S6. The ^{13}C-NMR spectrum of compound **2** (125 MHz, CD_3OD)

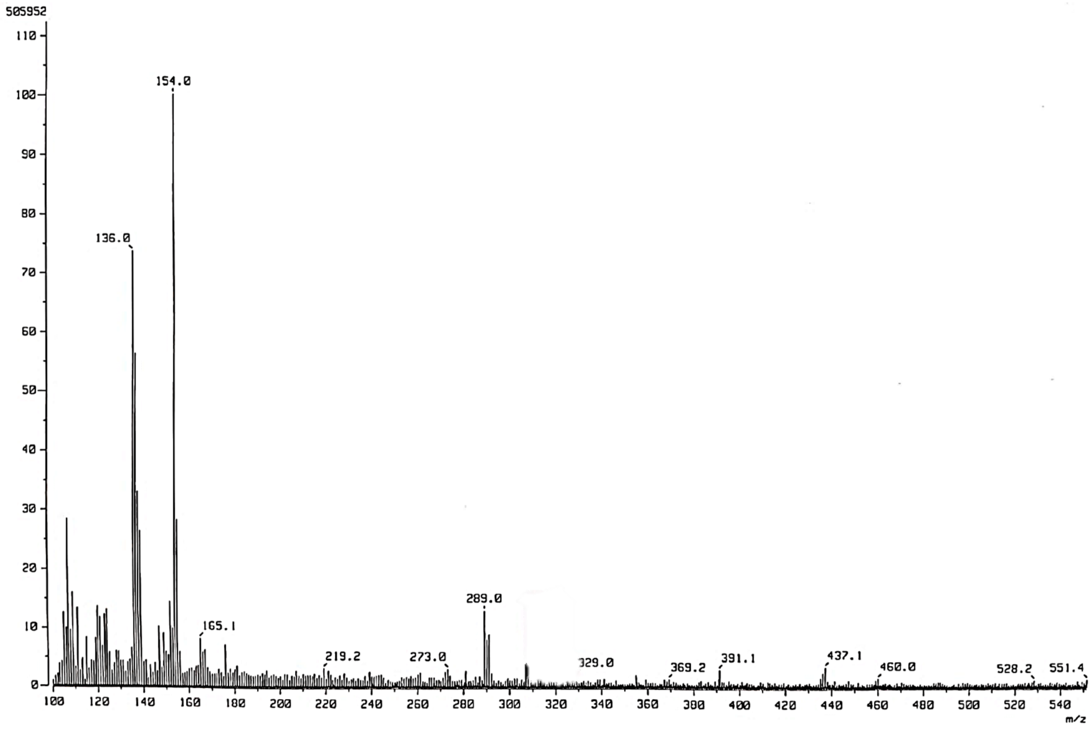

Figure S7. The FAB-MS spectrum of compound **3** (*m*-NBA)

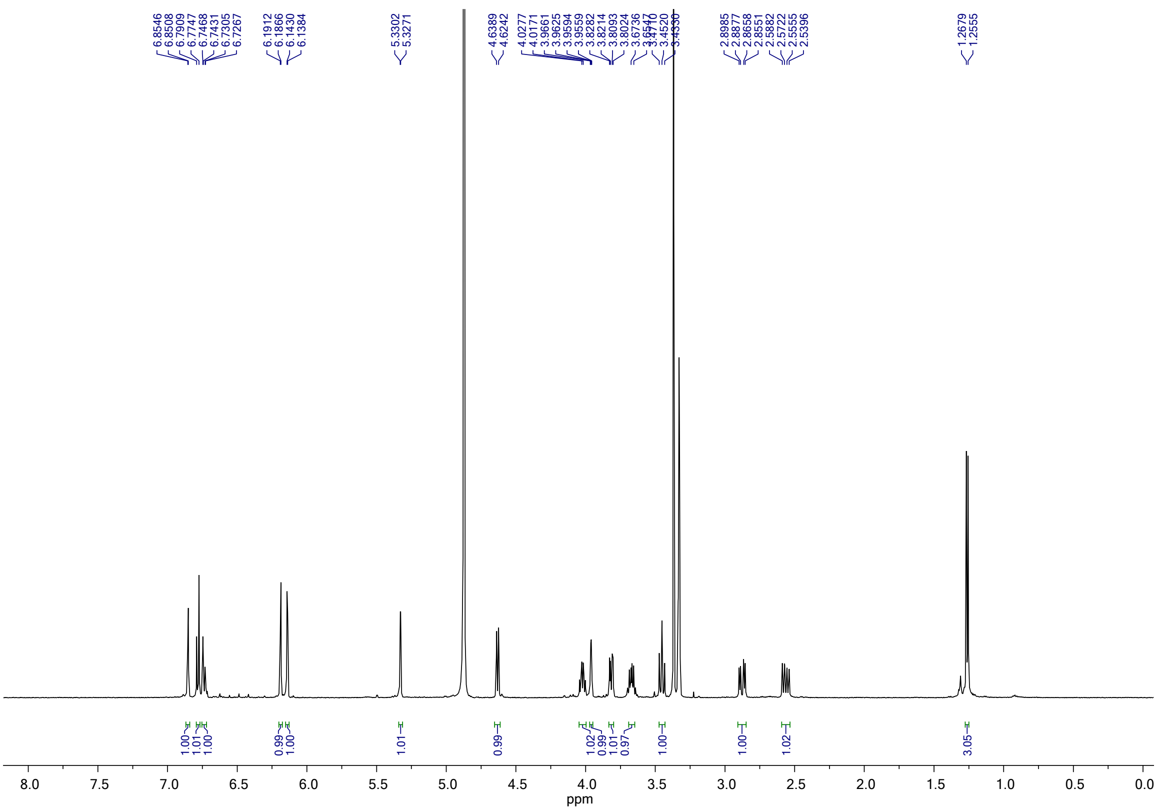

Figure S8. The ^{1}H-NMR spectrum of compound **3** (500 MHz, CD_3OD)

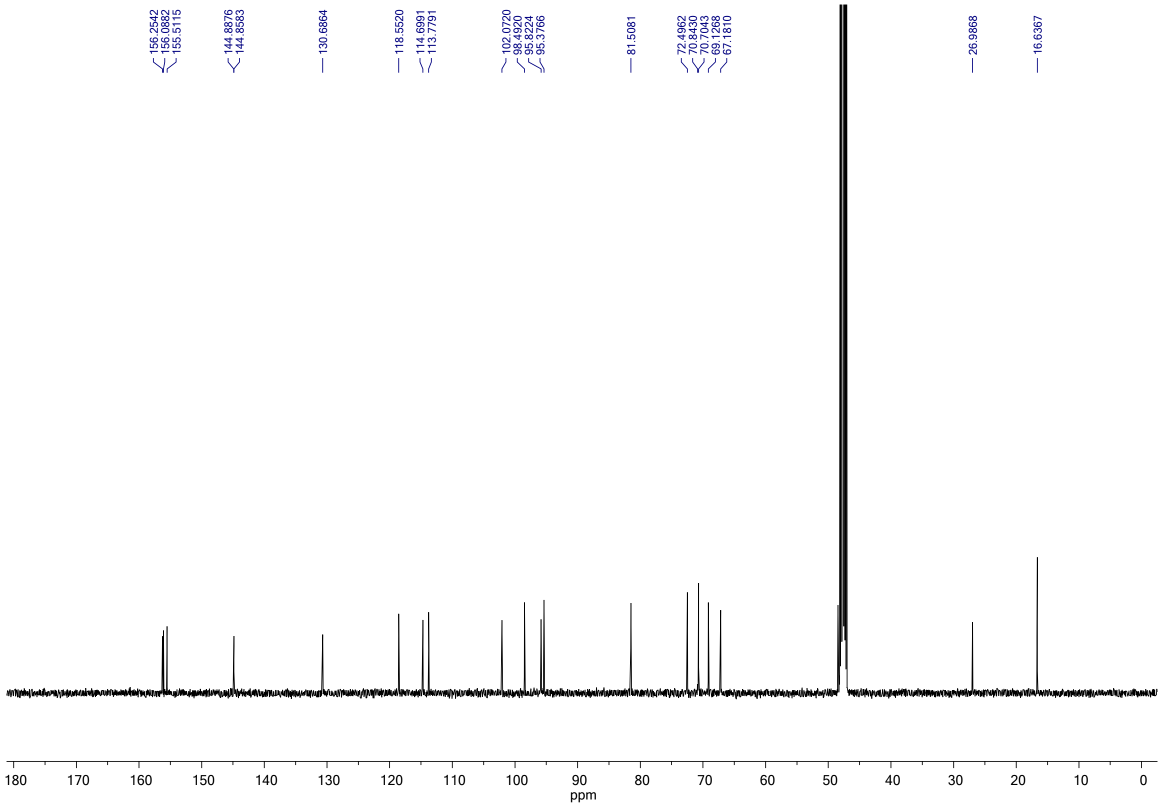

Figure S9. The ^{13}C-NMR spectrum of compound **3** (125 MHz, CD_3OD)

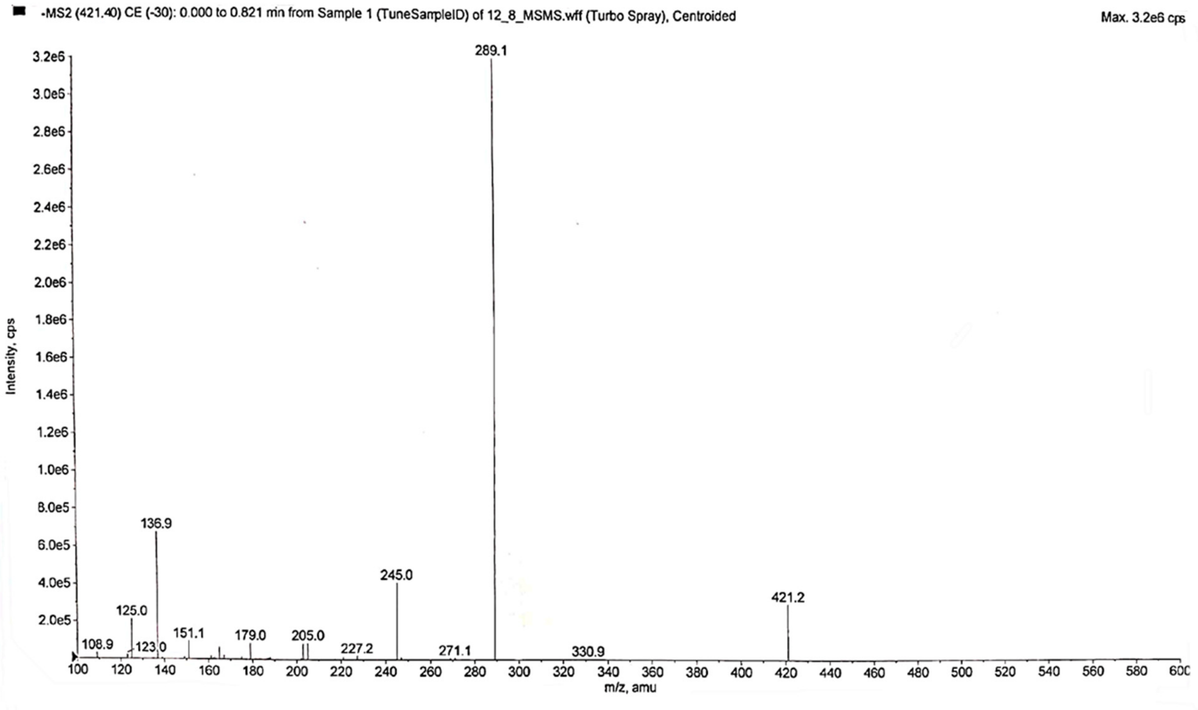

Figure S10. The ESI-MS spectrum of compound **4**

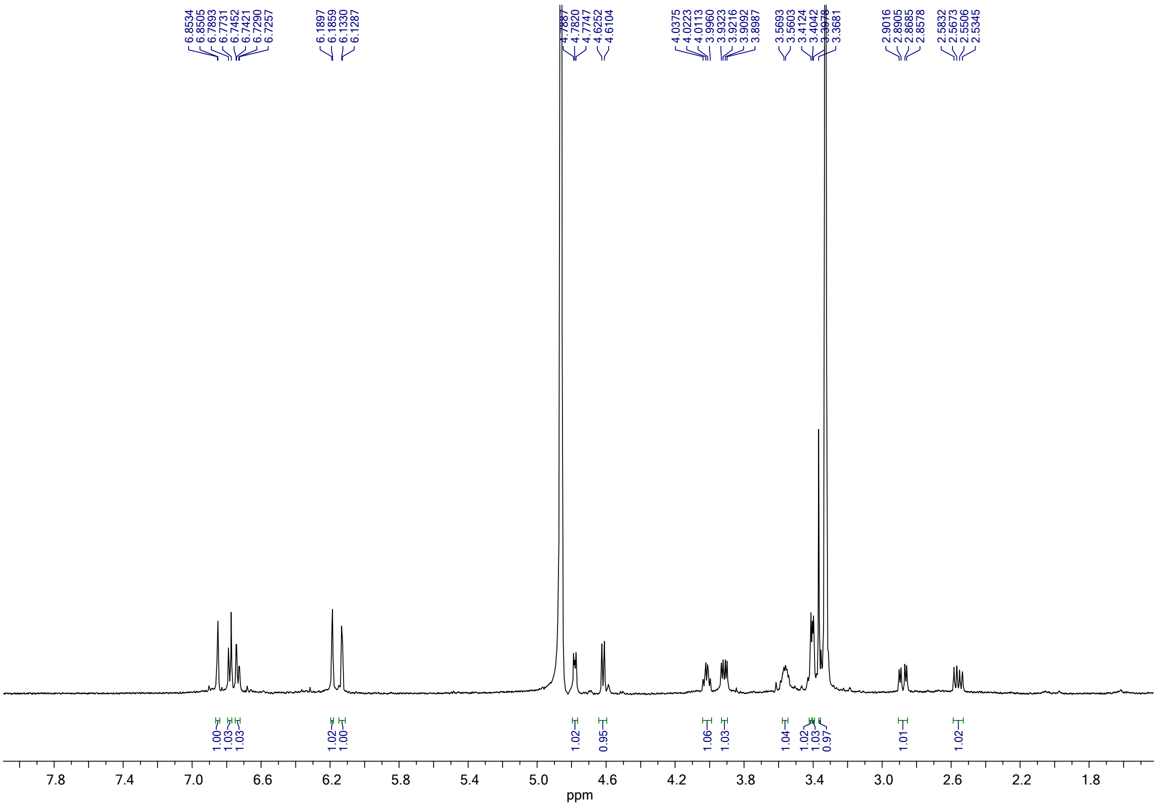

Figure S11. The ^{1}H-NMR spectrum of compound **4** (500 MHz, CD_3OD)

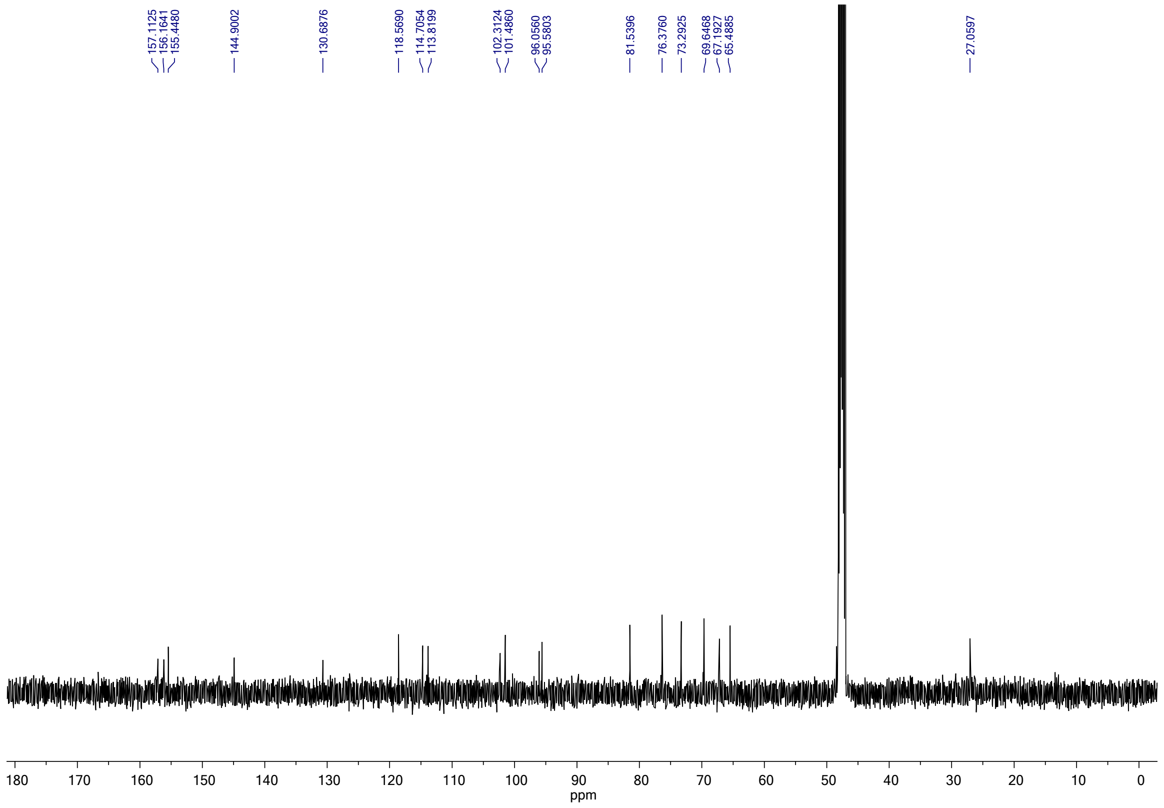

Figure S12. The ^{13}C-NMR spectrum of compound **4** (125 MHz, CD_3OD)

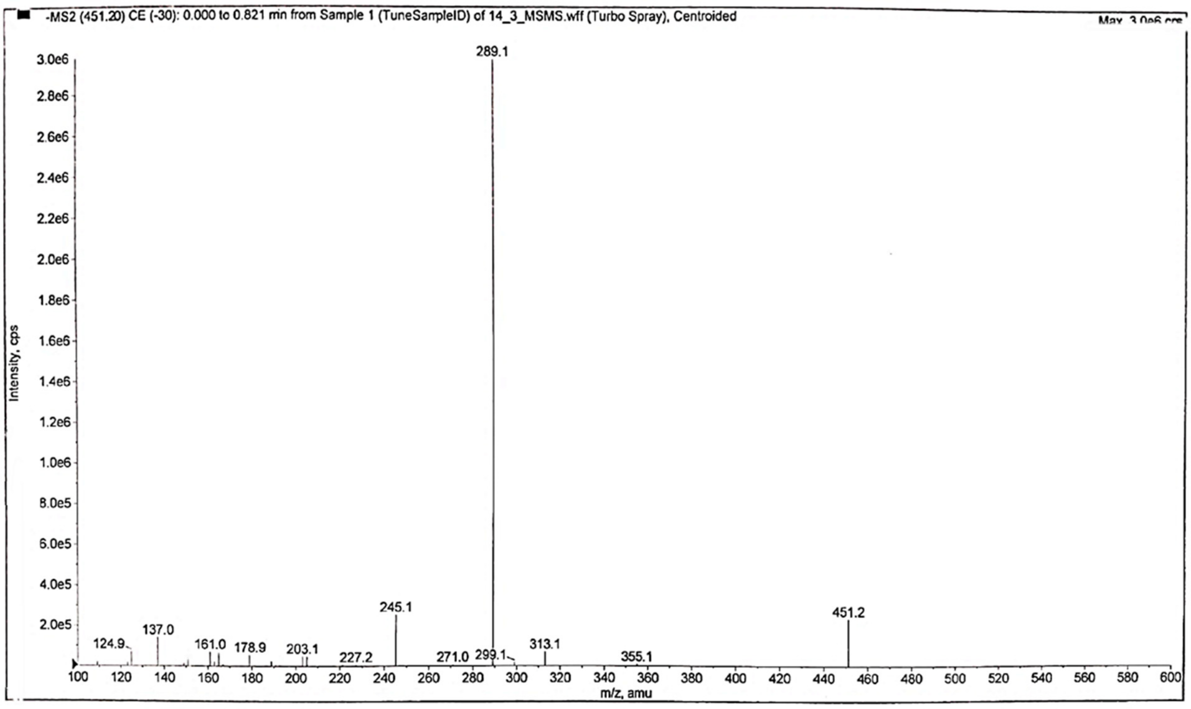

Figure S13. The ESI-MS spectrum of compound **5**

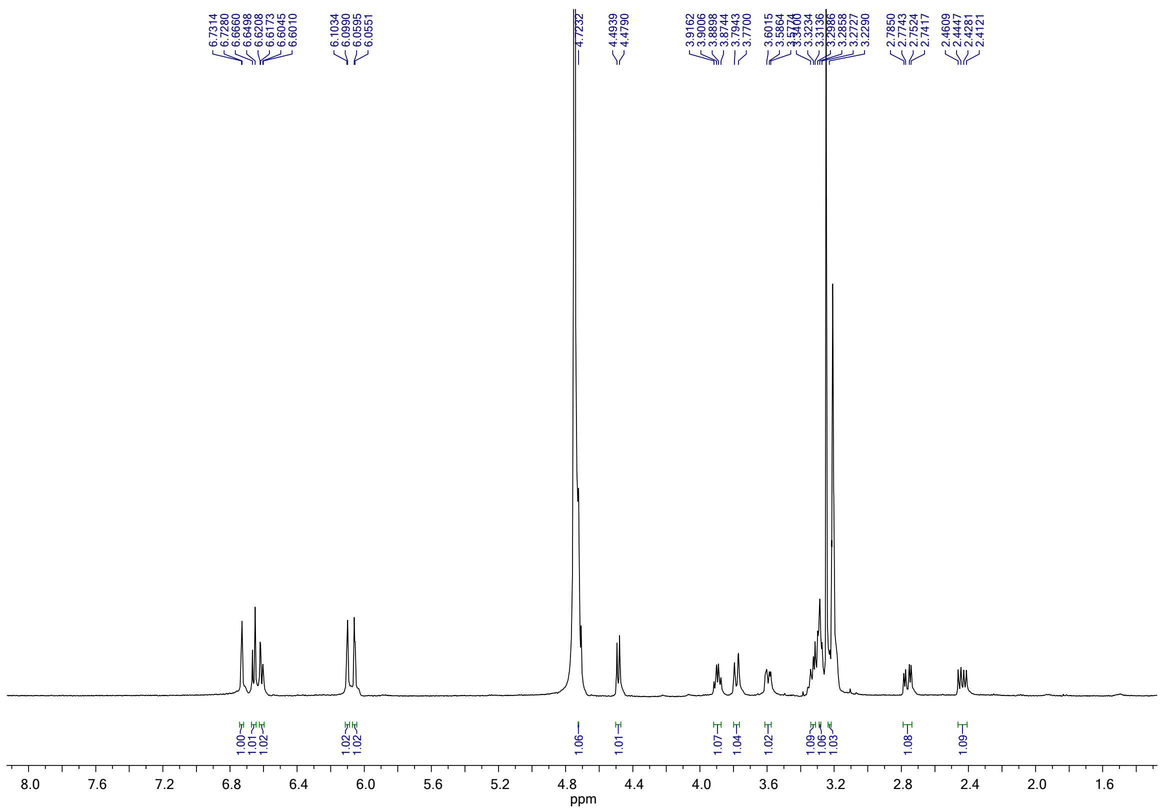

Figure S14. The ^{1}H-NMR spectrum of compound **5** (500 MHz, CD_3OD)

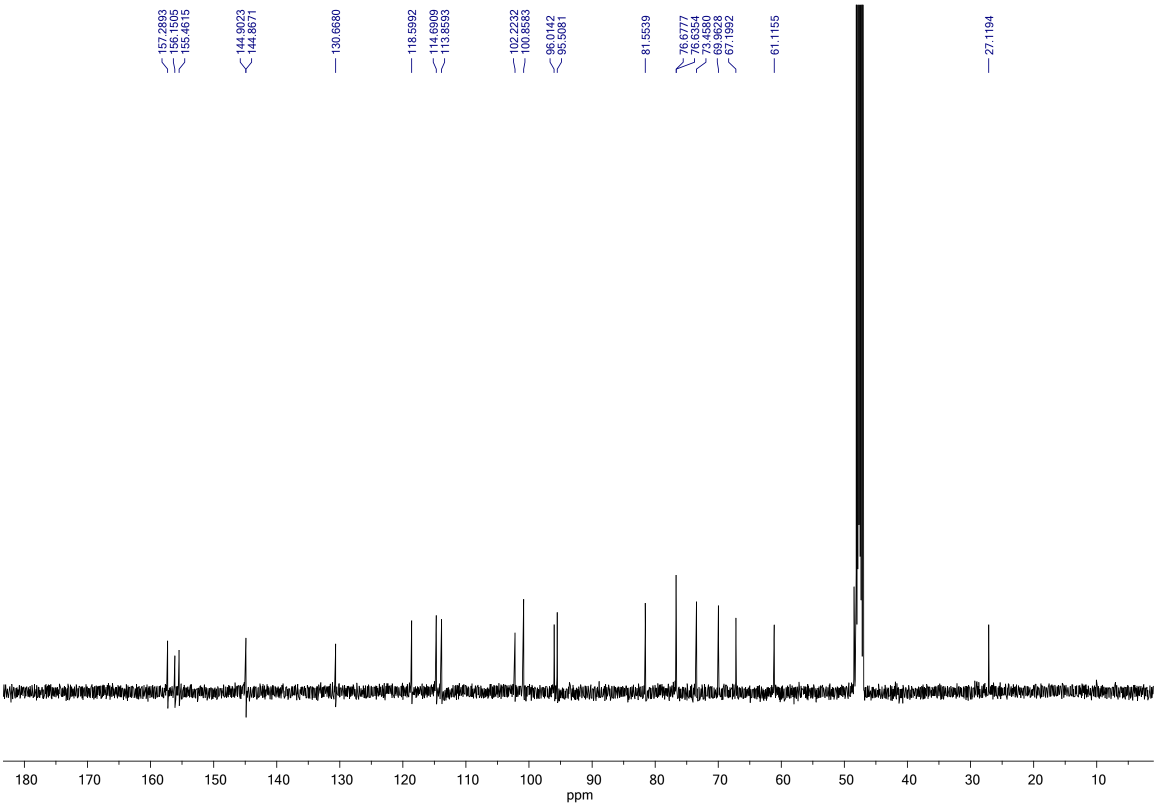

Figure S15. ^{13}C-NMR spectrum of compound **5** (125 MHz, CD_3OD)

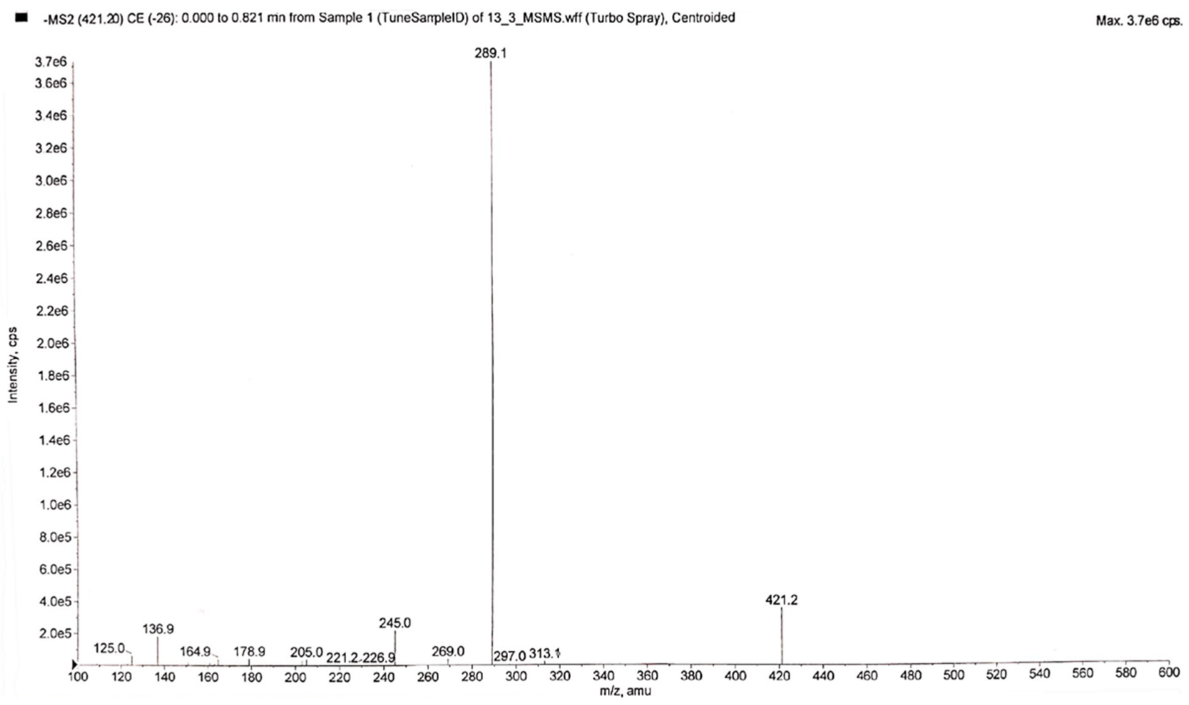

Figure S16. The ESI-MS spectrum of compound **6**

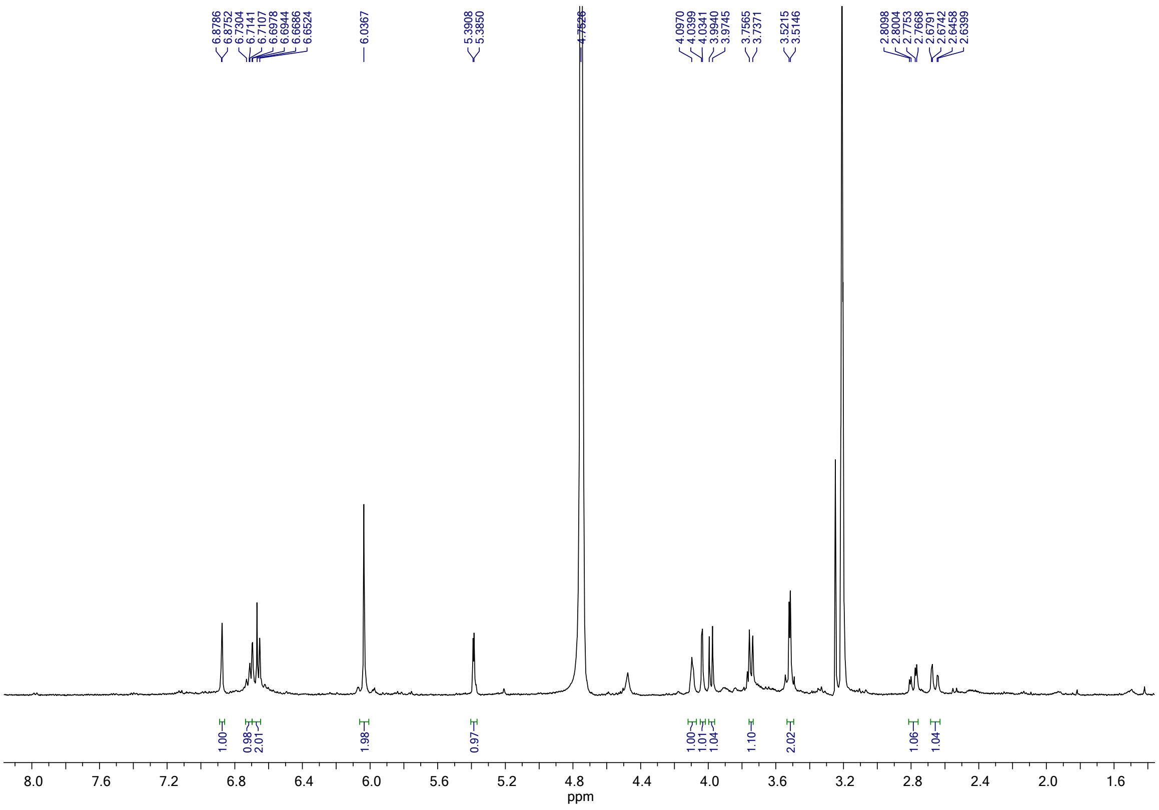

Figure S17. The ^{1}H-NMR spectrum of compound **6** (500 MHz, CD_3OD)

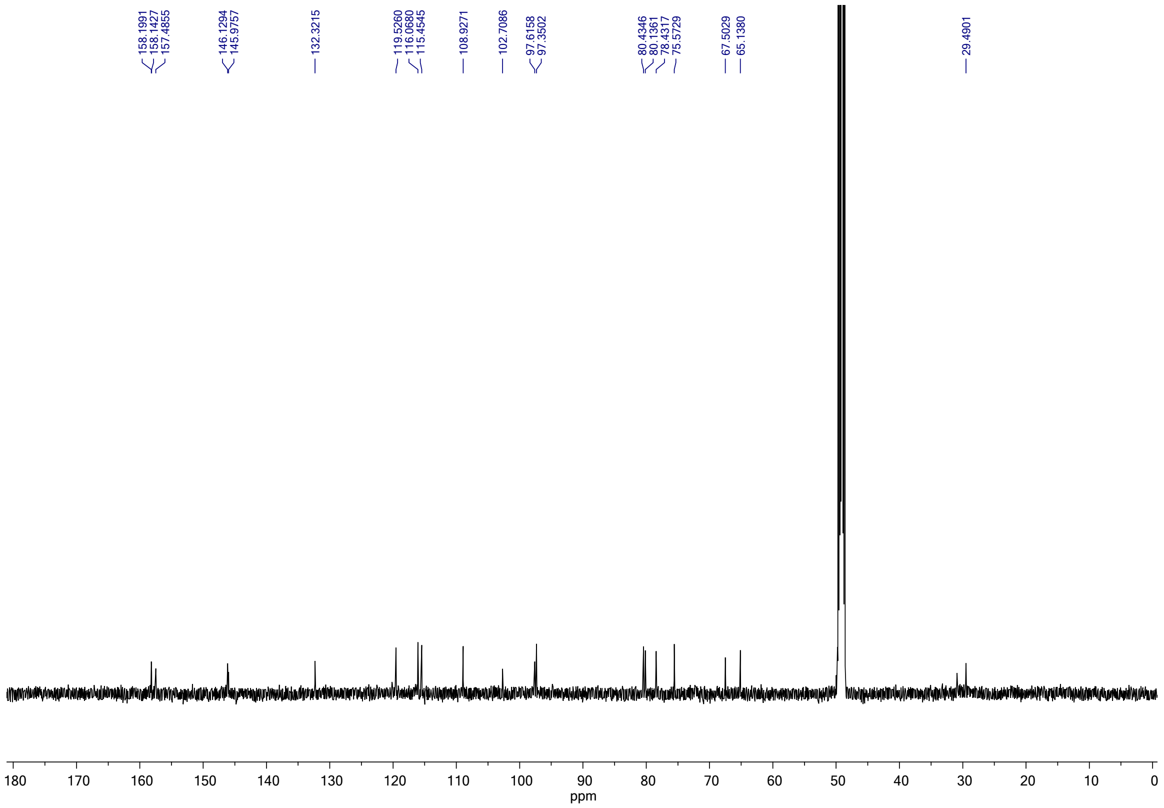

Figure S18. The ^{13}C-NMR spectrum of compound **6 (**125 MHz, CD_3OD)

^{1}H and ^{13}C NMR assign data of isolated compounds

(-)-Catechin (**1**): Pale yellow powder; $C_{15}H_{14}O_6$; EI-MS (*m/z*): 290 $[M]^+$; ^{1}H-NMR (CD_3OD, 300 MHz): δ 6.85 (1H, d, *J* = 1.9 Hz, H-2'), 6.78 (1H, d, *J* = 8.3 Hz, H-5'), 6.73 (1H, dd, *J* = 8.3, 1.9 Hz, H-6'), 5.94 (1H, d, *J* = 2.3 Hz, H-6), 5.87 (1H, d, *J* = 2.3 Hz, H-8), 4.58 (1H, d, *J* = 7.5 Hz, H-2), 4.01 (1H, ddd, *J* = 8.0, 7.5, 5.4 Hz, H-3), 2.87 (1H, dd, *J* = 16.4, 5.4 Hz, H-4eq), 2.55 (1H, dd, *J* = 16.4, 8.0 Hz, H-4ax); ^{13}C-NMR (CD_3OD, 125 MHz): δ 156.5 (C-7), 156.2 (C-5), 155.5 (C-9), 144.9 (C-4'), 144.8 (C-3'), 130.8 (C-1'), 118.6 (C-6'), 114.7 (C-5'), 113.9 (C-2'), 99.4 (C-10), 94.9 (C-6), 94.1 (C-8), 81.5 (C-2), 67.4 (C-3), 27.1 (C-4)

Catechin-7-*O*-*β*-D-apiofuranoside (2): Pale yellow powder; $C_{20}H_{22}O_{10}$; FAB-MS (*m/z*): 423.2 $[M+H]^+$; ^{1}H-NMR (CD_3OD, 300 MHz) : δ 6.85 (1H, d, *J* = 1.9 Hz, H-2'), 6.78 (1H, d, *J* = 8.3 Hz, H-5'), 6.73 (1H, dd, *J* = 8.3, 1.9 Hz, H-6'), 6.15 (1H, d, *J* = 3.4 Hz, H-6), 6.10 (1H, d, *J* = 3.4 Hz, H-8), 5.50 (1H, d, *J* = 3.0 Hz, H-1''), 4.62 (1H, d, *J* = 7.4 Hz, H-2), 4.15 (1H, d, *J* = 3.0 Hz, H-2''), 4.10 (1H, d, *J* = 9.7 Hz, H-4''a), 4.01 (1H, ddd, *J* = 8.0, 7.4, 5.4 Hz, H-3), 3.86 (1H, d, *J* = 9.7 Hz, H-4''b), 3.65 (1H, d, *J* = 11.5 Hz, H-5''a), 3.61 (1H, d, *J* = 11.5 Hz, H-5''b), 2.87 (1H, dd, *J* = 16.4, 5.4 Hz, H-4eq), 2.55 (1H, dd, *J* = 16.4, 8.0 Hz, H-4ax); ^{13}C-NMR (CD_3OD, 125 MHz) : δ 156.8 (C-7), 156.2 (C-5), 155.5 (C-9), 144.9 (C-3', 4'), 130.7 (C-1'), 118.6 (C-6'), 114.7 (C-5'), 113.8 (C-2'), 107.3 (C-1''), 101.8 (C-10), 95.9 (C-6), 95.4 (C-8), 81.5 (C-2), 78.9 (C-3''), 76.9 (C-2''), 74.0 (C-4''), 67.2 (C-3), 63.5 (C-5''), 27.0 (C-4)

Catechin-7-*O*-*α*-L-rhamnopyranoside (**3**): Pale yellow powder; $C_{21}H_{24}O_{10}$; FAB-MS (*m/z*): 437.1 $[M+H]^+$; ^{1}H-NMR (CD_3OD, 500 MHz) : δ 6.85 (1H, d, *J* = 1.9 Hz, H-2'), 6.78 (1H, d, *J* = 8.2 Hz, H-5'), 6.73 (1H, dd, *J* = 8.2, 1.9 Hz, H-6'), 6.19 (1H, d, *J* = 2.3 Hz, H-8), 6.14 (1H, d, *J* = 2.3 Hz, H-6), 5.33 (1H, d, *J* = 1.5 Hz, H-1''), 4.63 (1H, d, *J* = 7.3 Hz, H-2), 4.01 (1H, ddd, *J* = 8.6, 7.3, 5.3 Hz, H-3), 3.96 (1H, d, *J* = 1.5 Hz, H-2''), 3.82 (1H, dd, *J* = 9.4, 6.1 Hz, H-3''), 3.66 (1H, m, H-4''), 3.45 (1H, t, *J* = 9.5 Hz, H-5''), 2.88 (1H, dd, *J* = 16.4, 5.3 Hz, H-4eq), 2.56 (1H, dd, *J* = 16.4, 8.6 Hz, H-4ax), 1.26 (3H, *J* = 6.3 Hz, 5''-CH_3); ^{13}C-NMR (CD_3OD, 125 MHz) : δ 156.5 (C-7), 156.1 (C-5), 155.5 (C-9), 144.9 (C-3', 4'), 130.7 (C-1'), 118.6 (C-6'), 114.7 (C-5'), 113.8 (C-2'), 102.1 (C-10), 98.5 (C-1''), 95.8 (C-6), 95.4 (C-8), 81.5 (C-2), 72.5 (C-5''), 70.9 (C-3''), 70.7 (C-2''),69.1 (C-4''), 67.2 (C-3), 27.0 (C-4), 16.6 (5''-CH_3)

Catechin-7-*O*-*β*-D-xylopyranoside (4): Pale yellow powder; $C_{20}H_{22}O_{10}$; ESI-MS (*m/z*): 421.2 $[M-H]^-$; ^{1}H-NMR (CD_3OD, 500 MHz) : δ 6.85 (1H, d, *J* = 1.5 Hz, H-2'), 6.78 (1H, d, *J* = 8.0 Hz, H-5'), 6.73 (1H, dd, *J* = 8.0, 1.5 Hz, H-6'), 6.19 (1H, d, *J* = 2.1 Hz, H-8), 6.13 (1H, d, *J* = 2.1 Hz, H-6), 4.78 (1H, d, *J* = 7.0 Hz, H-1''), 4.62 (1H, d, *J* = 7.4 Hz, H-2), 4.01 (1H, ddd, *J* = 8.0, 7.4, 5.6 Hz, H-3), 3.92 (1H, dd, *J* = 6.2, 5.4 Hz, H-5''a), 3.57 (1H, m, H-4''), 3.41 (1H, m, H-3''), 3.40 (1H, m, H-2''), 3.36 (1H, m, H-5''b), 2.88 (1H, dd, *J* = 16.6, 5.6 Hz, H-4eq), 2.56 (1H, dd, *J* = 16.6, 8.0 Hz, H-4ax); ^{13}C-NMR (CD_3OD, 125 MHz) : δ 157.1 (C-7), 156.1 (C-5), 155.5 (C-9), 144.9 (C-3', 4'), 130.7 (C-1'), 118.6 (C-6'), 114.7 (C-5'), 113.8 (C-2'), 102.3 (C-10), 101.5 (C-1''), 96.1 (C-6), 95.6 (C-8), 81.5 (C-2), 76.4 (C-3''), 73.3 (C-2''), 69.7 (C-4''), 65.5 (C-3), 65.5 (C-5''), 27.1 (C-4)

Catechin-7-*O*-*β*-D-glucopyranoside (5): Pale yellow powder; $C_{21}H_{24}O_{11}$; ESI-MS (*m/z*): 451.2 $[M-H]^-$; ^{1}H-NMR (CD_3OD, 500 MHz) : δ 6.73 (1H, d, *J* = 1.3 Hz, H-2'), 6.66 (1H, d, *J* = 8.1 Hz, H-5'), 6.61 (1H, dd, *J* = 8.1, 1.3 Hz, H-6'), 6.10 (1H, d, *J* = 2.2 Hz, H-6), 6.06 (1H, d, *J* = 2.2 Hz, H-8), 4.72 (1H, d, *J* = 7.9 Hz, H-1''), 4.49 (1H, d, *J* = 7.5 Hz, H-2), 3.90 (1H, ddd, *J* = 8.0, 7.9, 5.4 Hz, H-3), 3.78 (1H, d, *J* = 12.0 Hz, H-6''b), 3.59 (1H, dd, *J* = 12.0, 5.5 Hz, H-6''a), 3.32 (1H, m, H-3''), 3.29 (1H, m, H-2''), 3.23 (1H, m, H-4''), 2.76 (1H, dd, *J* = 16.3, 5.4 Hz, H-4eq), 2.44 (1H, dd, *J* = 16.3, 8.0 Hz, H-4ax); ^{13}C-NMR (CD_3OD, 125 MHz) : δ 157.3 (C-7), 156.2 (C-5), 155.5 (C-9), 144.9 (C-3', 4'), 130.7 (C-1'), 118.6 (C-6'), 114.7 (C-5'), 113.9 (C-2'), 102.2 (C-10), 100.9 (C-1''), 96.0 (C-6), 95.5 (C-8), 81.6 (C-2), 76.7 (C-3'', 5''), 735. (C-2''), 70.0 (C-4''), 67.4 (C-3), 61.1 (C-6''), 27.1 (C-4)

Catechin-5-*O*-*β*-D-apiofuranoside (**6**): pale yellowish powder; $C_{22}H_{22}O_{11}$; ESI-MS (*m/z*): 421.2 $[M-H]^-$; ^1H-NMR (CD_3OD, 500 MHz) : δ 6.88 (1H, d, *J* = 1.7 Hz, H-2'), 6.70 (1H, d, *J* = 8.1, 1.7 Hz, H-6'), 6.66 (1H, dd, *J* = 8.1 Hz, H-5'), 6.04 (2H, s, H-6, 8), 5.39 (1H, d, *J* = 2.9 Hz, H-1''), 4.75 (1H, br s, H-2), 4.10 (1H, m, H-3), 4.04 (1H, d, *J* = 3.0 Hz, H-2''), 3.98 (1H, d, *J* = 9.7 Hz, H-4''a), 3.75 (1H, d, *J* = 9.7 Hz, H-4''b), 3.53 (1H, d, *J* = 11.4 Hz, H-5''a), 3.50 (1H, d, *J* = 11.4 Hz, H-5''b), 2.79 (1H, dd, *J* = 16.7, 4.7 Hz, H-4eq), 2.68 (1H, dd, *J* = 16.7, 2.5 Hz, H-4ax); ^{13}C-NMR (CD_3OD, 125 MHz) : δ 158.2 (C-5, 9), 157.5 (C-7), 146.1 (C-4'), 146.0 (C-3'), 132.3 (C-1'), 119.5 (C-6'), 116.1 (C-5'), 115.5 (C-2'), 108.9 (C-1''), 102.7 (C-10), 97.6 (C-6), 97.4 (C-8), 80.4 (C-2), 80.1 (C-3''), 78.4 (C-2''), 75.6 (C-4''), 67.5 (C-3), 65.1 (C-5''), 29.5 (C-4)
